# Nuclear Structure and its Modifications in Tumours

**DOI:** 10.1038/bjc.1954.9

**Published:** 1954-03

**Authors:** R. J. Ludford

## Abstract

**Images:**


### NUCLEAR STRUCTURE AND ITS MODIFICATIONS IN TUMOURS.

R. J. LUDFORD*

*From Mount Vernon Hospital, Northwood, Middlesex.*

Received for publication December 15, 1953.

IN an attempt to interpret the extraordinarily complicated structure often exhibited by malignant cells, a comparative study has been made of the nuclei of a wide range of tumours. Living cells have been examined by phase-contrast and ultra-violet microscopy, and sections and tissue cultures of tumours have been studied after fixation and staining by various histological and cytochemical techniques. From this survey has emerged a conception of nuclear structure and functioning which is at variance with that of the classical textbooks of cytology (Wilson, 1925), and conflicts with the views generally held to-day (Pollister, 1952).

*Technical considerations.*

Flattened cells have become increasingly employed for cytological research purposes. Modern techniques of microscopy, particularly phase-contrast, by greatly facilitating the study of living cells have resulted in greater use being made of tissue cultures for morphological studies. When cells migrate from an explant *in vitro*, they tend to spread out so that around the periphery of an outgrowth they are considerably flattened. This is most obvious in fluid culture media where the cells furthest away from the explant may be reduced to little more than a fine film. Such cells are ideal for the study of cytoplasmic structure, as many investigators have realised. Equally useful have proved squash preparations of soft tissues, when the cells of which they are composed tolerate compression without disrupting. The necessity of rapid fixation, in order to obtain perfect preservation of chromosome structure has also resulted in the common practice of using squash preparations and smears for the study of nuclear structures. Such procedures possess the further merit of eliminating the possibility of slight morphological alterations being brought about by the prolonged dehydrating, clearing and imbedding necessary for the preparation of sections. Nevertheless all these methods have the common disadvantage of rendering difficult the correct appreciation of the spatial arrangement of intranuclear structures. In this respect the older method of studying stained sections of fixed tissues presents definite advantages. By cutting sections of approximately the same thickness as the average diameter of the cell nuclei, one obtains preparations with whole nuclei and slices of nuclei of varying thickness. On examining such sections under the microscope there can be found superficial slices from the surface of nuclei, and sometimes a nucleus with the top of the membrane cut away making it possible to focus down into its interior. Often two constituent portions of a

* British Empire Cancer Campaign Senior Research Fellow.

nucleus can be detected in adjacent sections. Since no other technique offers the same scope for the direct examination of a three-dimensional structure, the study of stained sections has constituted the basis of this investigation. Yet with all fixed preparations of cells there arise doubts as to the validity of the finest structural details which are detectable, especially when they approach the limits of microscopic resolution. So the results of the study of fixed and stained cells have been correlated with observations made by other techniques, which have included the microscopical examination of living cells immediately after their removal from the animal body.

*Structural organization of the nucleus seen in sections.*

The ideal type of cell with which to commence an investigation of this kind would be one with a large nucleus, single nucleolus and well-defined chromatin granules. No tumour with such cells was available. Most nearly fulfilling these requirements were the cells of a mouse adenocarcinoma (T27). Its selection was further influenced by the fact that it had been employed in several previous researches (Ludford, 1924, 1930, 1932 *a*, 1932 *b*, 1933, 1934) so that the structure of its cells, and their behaviour under a variety of experimental conditions, as well as their growth characteristics, both *in vivo* and *in vitro*, were already well known.

The same methods of fixation and staining were adopted as were formerly used for the study of chromosomes (Ludford, 1930), except that after iron-alum-haematoxylin staining the differentiation was not carried so far as is usual in making chromosome preparations. Frozen sections stained by the Feulgen technique were also found most useful. For demonstrating the smallest structural details it is doubtful whether any method can excel the results obtained by Flemming fixation followed by intense staining with iron-alum-haematoxylin. When the surface of a nucleus from such a preparation is examined there are to be seen numerous minute granules varying in size. Most of them seem to be below the limits of microscopic resolution. They are depicted in the camera lucida drawings of Fig. 1 to 5. Fig. 1 shows the surface of a nucleus with a hole in the middle through which the nucleolus can be seen inside. Some of the little granules appear to be arranged like beads on a string. A slice off the surface of another nucleus from an elongated cell is shown in Fig. 2. There is a tendency for the granules to be elongated and orientated in the same direction as the cell is stretched. The holes appearing in both these first two figures were torn in the membranes in the process of section cutting. Fig. 4 depicts a slice through the centre of a nucleus. Here are seen the same peripheral granules, and also strands connecting the two nucleoli with the membrane. Along the length of the strands are granules of similar dimensions. Sometimes nucleoli are in direct contact with the nuclear membrane, as in Fig. 3, which is another slice from the surface of a nucleus. In this one, some well-defined strings of granules are discernible, while in the adjacent Fig. 5 all the granules are thus arranged. The largest are conspicuous at the ends of certain of the filaments. The latter obviously represent an early stage in the formation of the prophase chromosomes. Actually in a section of a rapidly growing tumour it is impossible to determine exactly when the prophase does begin. There are all transitions between scattered granules, rows of granules, and definite threads or filaments. The latter get progressively thicker, but the terminal knobs on some of them still remain clearly distinguishable (see also Fig. 44). A surface view and a section through the middle of a nucleus at prophase are represented in Fig. 6 and 7 respectively. Clearly most of the chromosomes lie just under the membrane, but a few are disposed around the nucleolus. Fig. 8 and 9 are sections through the middle of two other cells at prophase. In both nuclei there are the usual superficially arranged chromosomes with the interior nucleoli connected by strands to the

FIG. 1 to 5.—Nuclear structure of adenocarcinoma (27) cells seen in sections. Flemming fixation : iron-alum-haematoxylin staining.

FIG. 1.—Nucleus cut tangentially showing peripheral granules, representing chromosomes. Nucleolus seen through hole in the nuclear membrane.

FIG. 2.—Slice of nucleus with elongated peripheral particles on under surface of membrane.

FIG. 3.—Surface of a nucleus with small nucleolus directly underneath the membrane.

FIG. 4.—Section through a nucleus showing nucleoli connected to the periphery by strands bearing granules.

FIG. 5.—Early stage of chromosome formation on the under surface of the nuclear membrane.

periphery. In Fig. 9 these strands are double like the chromosomes at the surface. In Fig. 8 they appear as single threads, and granules are perceptible on some of them. It thus seems that the nucleoli are connected with the periphery of the nucleus by chromosomes, and also by threads of a different nature. Frequently the latter appear to be attached distally to a chromosome. Examination of prophases at the stage when the nuclear membrane is undergoing dissolution will sometime reveal chromosomes connected with remnants of nucleoli. A section through the middle of such a cell is depicted in Fig. 10. Adherent to two of the chromosomes are globular remains of nucleoli.

FIG. 6 to 12.—Nuclear structure of adenocarcinoma (27) cells seen in sections. Flemming fixation : iron-alum-haematoxylin staining.

FIG. 6.—Surface view of prophase nucleus with chromosomes on the under surface of the nuclear membrane.

FIG. 7.—Section of the same cell showing peripheral chromosomes and others around the nucleolus.

FIG. 8, 9.—Sections of prophase nuclei illustrating differences in the strands connecting the nucleoli with the nuclear membrane.

FIG. 10.—Section of cell at the time of dissolution of the nuclear membrane. Remnants of nucleolar material attached to two chromosomes.

FIG. 11, 12.—Karyomeres in cells which have resulted from a multipolar mitosis following irradiation. Two of the karyomeres in Fig. 12 exhibit the beginning of nucleolar formation in association with chromosomes.

The origin of the nucleolus at the reconstruction phase of mitosis is very difficult to determine, even in Feulgen preparations counterstained with a nucleolar staining dye. Though the cells are comparatively large for mammalian cells, they are not ideal material for studying this aspect of the problem. An attempt has, however, been made to gain an insight into what happens during the reconstruction phase by examining sections of tumours which had been irradiated *in vivo*. In an earlier investigation of the cytological changes following irradiation of this tumour (Ludford, 1932*a*) it was observed that the wave of cell division which supervened upon the initial inhibition was characterised by numerous aberrations of mitosis, which included multipolar figures, and these resulted in several daughter cells whose nuclei were represented by karyomeres formed by chromosome fragments, single chromosomes and groups of chromosomes. Search was made amongst such cells for the earliest stages of nucleolus formation. Fig. 11 and 12 show three of the cells resulting from a single mitosis. These two figures were drawn from consecutive sections. In Fig. 11 it will be noticed that the karyomeres consist of whole or fragmented chromosomes spread on the surface of vesicles. No nucleoli are present. Contrariwise, the two largest of the karyomeres in Fig. 12 each contain a small nucleolus attached to chromosomes. The frequent occurrence of chromosome fragments following irradiation makes it uncertain whether we are here concerned with whole, or parts of chromosomes. Nevertheless it is evident that both at prophase, and at the reconstruction phase of mitosis, certain chromosomes can be seen to be directly attached to nucleoli. This conclusion conforms with the view that the nucleolus is formed by the action of a specific region of particular chromosomes, which has been called the " nucleolar organizer ". For the literature on this subject reference should be made to the review of Gates (1942).

*Nuclear structure of cells grown* in vitro.

When living cells are compressed between slide and cover slip, or when they spread out in tissue cultures, their nuclei tend to become flattened like pancakes, thereby concealing the structural organization just described (Fig. 42 and 43). But once the latter has been recognised, flattened cells provide a favourable means of examining certain of its features. Fig. 13 represents part of a cell of the same carcinoma on the edge of a 2-days-old culture, which had been fixed in Flemming's fluid, and intensely stained with iron-alum-haematoxylin. The fine peripheral granules beneath the nuclear membrane which are shown in Fig. 1 to 5 have been omitted from Fig. 13 to 20. With the exception of Fig. 16 and 17, these are projection drawings (indicated by " P " in the legends). There are included in the plane of each drawing bodies distributed throughout the whole nucleus. Thus each drawing constitutes a diagrammatic representation of the structures as if they were projected on to the equatorial plane. In Fig. 13 the large central nucleolus is connected by very delicate threads to two granules underlying the nuclear membrane. A smaller nucleolus is directly in contact with the membrane. In addition there are six other granules. No connections can be detected between them and the nucleolus. This does not mean none is present, because extremely thin threads might only be visible when coated with dyestuff, and this may have been removed in the early stage of differentiating with the iron-alum. The nucleus seen in Fig. 14 is in a cell from an older culture (7 days) where degenerate changes are occurring. It appears to be undergoing amitotic division. Similar

**Fig. 13 to 20.—Nuclear structure of adenocarcinoma (27) cells seen in tissue cultures. Fig. 13 and 14, Flemming fixation : iron-alum-haematoxylin staining. Fig. 15, 16 and 17, Bouin-Allen fixation : iron-alum-haematoxylin staining. Fig. 18, 19 and 20, Corrosive-acetic fixation : Feulgen staining, without counterstaining. (P = projection drawing : see text.).**

**Fig. 13.—Cell from a 2-days-old culture, showing nucleolus connected by fine strands to peripheral granules (P).**

**Fig. 14.—Cell from a 7-days-old culture, showing emission of chromatin granules from the surface of the nucleoli (P).**

**Fig. 15.—Nucleolar system of a well-spread cell on margin of outgrowth, de-stained so that only nucleolar chromatin remains black (P).**

**Fig. 16—Surface view of nucleus with chromatin particles of nucleolar origin on under-surface of membrane.**

**Fig. 17.—Nucleus of same cell, containing intranucleolar vacuoles and granules. Little nucleolar chromatin remains on surface of plasmosome.**

**Fig. 18, 19 and 20.—Nuclei demonstrating variations in the distribution of deoxyribonucleic acid inside the nucleus. Nucleolus unstained. Nucleolar chromatin in the form of vesicles in Fig. 20 (P).**

delicate strands are visible connecting the nucleolus with the peripherally disposed granules or with the membrane. Each of the nucleoli is surrounded by particles which appear to have been emitted from its surface.

If observation is now extended to Feulgen-stained cultures it becomes evident that the nucleolus is of a dual nature. The greater part of it is Feulgen negative, but on its surface are irregular-shaped globules of Feulgen positive material (Fig. 18 to 20). The latter constitutes what Caspersson (1947) has termed " the nucleolar associated chromatin " and to which he attributes a special role in protein synthesis. It can be distinguished from the material of which the bulk of the nucleolus is composed in the living condition, and in fixed preparations after a variety of staining methods. Examined by phase-contrast microscopy in the live cell the " nucleolar chromatin", as it is proposed to call it, looks darker. The difference is more marked in ultra-violet micrographs taken with wave-lengths of 2570 Å and 2750 Å (Fig. 33, 35 and 37). It stains deeper with most dyestuffs, which stain the nucleolus. In tissue cultures fixed in Bouin-Allen fluid, it is possible to differentiate after iron-alum staining so that only the nucleolar chromatin retains the stain. This is demonstrated in Fig. 15. In the nucleus depicted here, the nucleolus is broken up into a number of bodies of varying sizes and shapes. Each bears deeply stained particles of chromatin. In neighbouring cells of the culture even the chromosomes of dividing cells are de-stained. On the basis of the reaction of the two nucleolar components to staining by the Feulgen and the pyronine-methyl-green techniques it is concluded that the chromatin is composed of appreciable amounts of deoxyribose nucleotides, and the remainder of the nucleolus of ribose nucleotides.

There is considerable variation in the amount of nucleolar chromatin in different cells, as is demonstrated by Fig. 18, 19 and 20. Here are nuclei drawn from cells of the same culture which was stained by the Feulgen method, without counterstaining. The bulk of each nucleolus, therefore, remains colourless. Sometimes the nucleolar chromatin appears to form vesicles, as is shown in Fig. 20.

In one form of cellular degeneration as has previously been mentioned, particles are discharged from the surface of the nucleolus. Comparison of cells stained by the iron-alum-haematoxylin technique, such as that shown in Fig. 14, with others stained by the Feulgen method reveals that the emitted particles are derived from the nucleolar chromatin. The same phenomenon is illustrated in Fig. 16 and 17 which are drawings made from the same nucleus. Fig. 16 shows its upper surface with chromatin particles of nucleolar origin. Fig. 17 is a median optical section, depicting what remains of the nucleolus. It contains vacuoles of varying sizes and minute granules of different shapes. The latter are stained by haematoxylin, but are invisible in Feulgen preparations. There are also distinguishable in nucleoli varying numbers of argentophile bodies. Page, Regan and MacCarty (1938) found that both types of inclusions were more numerous in malignant than in non-malignant cells, and that " the more malignant the neoplasm, the greater the number of intra-nucleolar bodies."

Examination of nucleoli with the electron microscope has suggested the existence of a more complicated structure. Thus Borysko and Bang (1952) have described a complex nucleolar structure, usually consisting of a tangled mass of branching or anastomosing filaments, approximately 0·1 micron in width. No suggestion of anything comparable was apparent in any of the living cells, or in the fixed and stained ones that were studied in the course of this work.

It must, however, be conceded that the microscopical techniques employed in the present work did not give the high resolution which is obtained with the electron microscope. The limit of resolution attained with the ultra-violet microscope is only about 0·1 micron. This is the best resolution represented in the figures illustrating this paper.

Occasionally when a nucleolus is highly vacuolated its appearance is rather deceptive, and might erroneously be interpreted as being reticulate. One wonders whether the complicated internal structure which has been described may be the result of a slight distortion induced by the procedures involved in preparing sections for examination by the electron microscope. The vacuolated appearance is not an artefact of fixation as it is visible in living cells.

*Distribution of nucleic acids in the nucleus.*

As a matter of convenience, for the purpose of description, it is proposed to refer to the material of which the nucleolus is mainly composed as " plasmosomin". This is the material which constitutes "the true nucleolus or plasmosome " (Wilson, 1925). The term has a purely morphological denotation, but connotates one chemical distinction. The nucleolar chromatin contains sufficient deoxyribose nucleotides to stain intensely by the Feulgen technique, while plasmosomin does not. This does not eliminate the possibility that in the latter deoxyribose nucleotides may be present in such small quantities as to be below the limit of sensitivity of the Feulgen technique, upon the validity of which as a test for deoxyribonucleic acid this only definitely known chemical difference between nucleolar chromatin and plasmosomin depends.

Cytogeneticists find it convenient to use the terms " euchromatin " and " heterochromatin". Their usage is based upon the conclusion that at the end of mitosis as the chromosomes uncoil deoxyribonucleic acid remains attached at certain loci (heterochromatin), and is discharged from the rest of the chromosome (euchromatin). Since it has been impossible to see whether this actually happens in the cells employed in this investigation no attempt has been made to distinguish between two types of chromatin. The term " chromatin " is used in this paper to describe nuclear material which gives a positive reaction with the Feulgen test, and which is not visibly connected with the nucleolus. In the latter case it is called nucleolar chromatin.

Whether one studies fixed and stained Feulgen preparations, or ultra-violet micrographs of living cells, it becomes obvious that there must be an increase of deoxyribonucleic acid at prophase, and a corresponding decrease during the reconstruction phase of mitosis. Evidence has already been adduced that the chromosomes are represented in the " resting " nucleus by the fine granules at its surface, and on strands connecting the nucleolus with the nuclear membrane. These granules appear to be faintly stained by the Feulgen technique. But it is doubtful whether any definite conclusion can be justifiably drawn from pale staining, as it has been pointed out, most of these minute particles are just above, or below, the limits of microscopic resolution. If they fail to stain that does not necessarily mean that they contain no deoxyribonucleic acid. It might well be that the amounts present in such small particles would be undetectable by the technique. Also very faint staining could result from absorption of traces of diffuse dyestuff. Our present microscopical methods are incapable of supplying unequivocal evidence which could finally solve such problems.

Similar considerations are involved in the problem of the nucleic acid content of the nuclear sap. The latter usually appears to be very faintly coloured in Feulgen preparations, and the nuclear periphery is always most clearly defined even in the absence of cytoplasmic counterstaining. There is by comparison an intense staining of larger peripheral particles, and of others on the filaments connecting the nucleoli with one another and with the nuclear membrane. Ultra-violet micrographs of living cells exhibit a very distinct nuclear membrane (Fig. 35) which often presents the appearance of a narrow hyaline layer (Fig. 36 and 37) with particles of strongly absorbing material applied to its inner surface (Ludford and Smiles, 1950*b*). The fluid filling the interstices of the nuclei is responsible for a slight diffuse absorption. This disappears at the late prophase when the chromosomes are fully formed. The clearer nuclear sap at this stage is also obvious in fixed and stained sections when examination is carried out with the light microscope. Before discussing the possible significance of these observations attention will be directed to some relevant observations on the state of the nucleus following destruction of the cytoplasm.

*Structure of the gelated nucleus.*

Micrurgical experiments performed on mammalian tumour cells have failed to elucidate any difference between them and their normal prototypes (Chambers and Ludford, 1932). In all cases, puncture of the nucleus results in its collapse and irreversible coagulation. Tearing the cell membrane produces a similar result, but gently puncturing the cytoplasm does not.

By compressing small fragments of tumour between slide and cover slip the cytoplasm can often be destroyed without disrupting all the nuclei. Those which remain intact after this treatment become more conspicuous by transmitted light owing to their gelated condition. With dark-ground illumination they appear opalescent, which is a characteristic feature of dead cells (Ludford, 1935). Fig. 30 is an ultra-violet micrograph of some flattened coagulated nuclei from another mouse carcinoma (Af). It will be noticed that the superficial structure is very similar to that drawn in Fig. 2. In both illustrations there are granules, rows of granules and threads or filaments. The nucleoli are also visible in Fig. 30, but are not in focus, since the ultra-violet microscope has a very limited depth of focus. In some instances the strings of granules appear to be double with droplets between them. Smearing the nuclei accentuates this arrangement of parallel rows of granules separated by a hyaline material, as is demonstrated in Fig. 31. Here the remains of nucleoli are also still distinguishable. The appearances presented by Fig. 30 and 31 are suggestive of the ultra-structure of a gel in the unstretched state (Fig. 30) when it is a tangle of long chain molecules, and in the stretched state (Fig. 31) when the molecules have become orientated in a direction parallel to the stretching force (Moyer, 1942).

The filamentous nuclear structure is reminiscent of that described in nuclei of spermatocytes of the grasshopper (*Dissosteira*) by Chambers (1924). He pointed out that in these nuclei, changes are taking place preparatory to the appearance of the meiotic chromosomes. Within a minute of pricking the surface of such a nucleus with a microdissection needle " hazy streaks of granules " appear. They increase in number and in size, and "the filamentous structure slowly thickens until one begins to appreciate that the body of the filament consists of a hyaline core with granules adherent to its surface". Although in side view the filaments appear double, Chambers, (1924) stated that in optical section " they are cylindrical with the groups of granules arranged about a hyaline core." The filaments shorten and thicken, and simultaneously the granules fuse together. In this way the chromosomes are formed. Chambers concluded that the structures revealed by nuclear puncture were not formed *de novo* as the result of the injury. They were invisible before owing to the uniformity of the refractive index of the nuclear contents.

The readiness with which nuclei coagulate is correlated with their high content of nucleic acids. Bensley (1938) isolated a substance from hepatic cells which he named " Plasmosin " and regarded as the gel- and fibre-forming constituent of protoplasm. The work of Mirsky and Pollister (1943) led them to the conclusion that this was a fibrous protein composed of histone and deoxyribonucleic acid, which was localised largely if not entirely inside nuclei. Hoerr (1943) does not agree that plasmosin is confined to nuclei, and has given reasons for believing that considerable amounts are present in the cytoplasm of liver cells. Other investigators have also reported the presence of deoxyribonucleic acid in the cytoplasm of plant cells (Sparrow and Hammond, 1947 ; Chayen and Norris, 1953). In the tumour cells which have been employed in the present work it has not been possible to detect any Feulgen positive material in the cytoplasm, and that stained deeply in the nucleus is sometimes small in amount (Fig. 18). The necessity for caution in interpreting the results of staining reactions has already been stressed.

Relevant to the present considerations is the work which has been carried out with the products of nuclear disintegration—the so-called " chromatin threads " (Claude and Potter, 1943 : Mirsky and Pollister, 1943). They stain by the Feulgen technique and are frequently paired. They have been regarded as the chromosomes, or fragments of chromosomes, of the interphase nucleus, but this interpretation of their nature has been disputed by Lamb (1950). It is highly probable that they are identical with the strands of material exhibiting strong absorption of ultra-violet radiations of 2570 Å wave-length, which are seen in Fig. 31. The absorbing material presumably comprises the fine granules, which for reasons already stated are regarded as representing the chromosomes of the interphase nucleus. Probably their volume is augmented at the time of nuclear coagulation as the result of the accretion of additional deoxyribonucleic acid from the nuclear sap, by a process analogous to that which occurs at the prophase of mitosis.

*Relationship between nucleolar chromatin and plasmosomin.*

On the basis of the preceding considerations it is concluded that the highest concentration of deoxyribonucleic acid is in the nucleolar chromatin and bodies derived from it. Deoxyribonucleic acid is also present in the minute granules which represent the interphase chromosomes and in the nuclear sap. Considerable evidence has been adduced by other investigators to prove that the plasmosome is particularly rich in oxyribonucleic acid.

The functional relationship between nucleolar chromatin and plasmosomin requires further investigation. Both are composed of nucleoproteins although of a different type, and are presumably as complex chemically as the other cell organs. The observations that are reported in this paper are consistent with two possibilities. Either nucleolar chromatin and plasmosomin are built up from the same simple precursors, and there are alternate pathways of metabolism the course of which can be deflected so as to lead to the formation of either one or the other substance. Or, there are contained in plasmosomin enzyme systems responsible for the synthesis of the substances comprising nucleolar chromatin from certain of its chemically simpler constituents; or perhaps for breaking down plasmosomin and converting it into nucleolar chromatin. The latter notion finds support from the behaviour of the nucleolus during the formation of the chromosomes at prophase. Fig. 36 and 37 are ultra-violet micrographs of living prophase nuclei of a sarcoma (Rb). In the former the surface of the nucleus is in focus and the peripheral chromosomes are seen in process of formation. In the latter the nucleoli in the interior of the nucleus are in focus. The darker nucleolar chromatin is distinguishable from the lighter plasmosomin. The nucleoli are in process of disintegration, and there is a considerable production of nucleolar chromatin. After the chromosomes are fully formed, small spherical masses of plasmosomin are often distinguishable devoid of any chromatin. At this stage all the Feulgen positive material has been deposited on the chromosomes. The gradual diminution in the amount of plasmosomin during the prophase does not mean that it is all being converted into chromatin as a considerable part of it is, presumably, incorporated in the chromosomes in the form of ribonucleoprotein.

*The nucleolar system of malignant cells and their non-malignant prototypes.*

By the nucleolar system is meant here the total nucleolar content of a nucleus, whether it be concentrated in a single structure, or dispersed as a number of smaller interconnected bodies occupying the greater part of the interior of the nucleus. In the latter case it is usual for each discrete portion to comprise plasmosomin and nucleolar chromatin. Parts of nuclei of a mammary carcinoma (Af) are shown in the ultra-violet micrographs of Fig. 32 and 33. In the former the nuclear surface is in focus, and in the latter the underlying nucleolar bodies are clearly defined. In each of them darker peripheral globules of nucleolar chromatin are distinguishable from the lighter plasmosomin.

While hypertrophy of the nucleolar system is a common feature of tumour cells, it is not a diagnostic characteristic of malignancy. The nuclear structure of cells of spontaneous mammary carcinomata of high-cancer-strain mice is remarkably like that of mammary gland cells. The similarity is particularly striking when both kinds of cells are grown under identical conditions in tissue cultures, as will be appreciated by comparing Fig. 38 and 39. The former was photographed from a 3-days-old primary culture of mammary carcinoma (Ad) in A strain mice ; the latter from a 5-days-old primary culture of mammary gland from a low-cancer-strain mouse (C57). Of more than 20 spontaneous mammary carcinomata which have been grown *in vitro* and examined cytologically the cells of the tumour illustrated in Fig. 38 exhibited the greatest degree of polymorphism. Note also in this figure the derangement of mitosis which has resulted in two daughter nuclei with numerous karyomeres instead of nuclei.

Despite the similarity of the nuclear structure of the cells of Fig. 38 and 39 their behaviour *in vitro* differs. Explants of carcinomata give rise to sheet growths of cells with greater regularity and rapidity than do those of mammary glands. They exhibit more mitoses, and more mitotic aberrations. Their cytoplasm does not usually contain so many fat droplets as does that of mammary gland cells. In general, spontaneous mammary carcinomata are easy to grow *in vitro*, while mammary gland is difficult.

It should be pointed out that the mammary carcinomata referred to here all originated spontaneously in high-breast-cancer-strain mice. Three factors are involved in their aetiology, namely, genetic constitution, hormonal stimulation, and the milk factor, or virus of Bittner (1951). The cells of other mammary cancers induced by carcinogenic compounds, independently of the milk factor, exhibit greater abnormalities, which distinguish them from normal mammary gland cells. (Kirschbaum and Bittner, 1945).

There is corresponding variation in the extent to which sarcoma cells differ from fibroblasts with respect to nuclear structure. The nucleolar system of fibroblasts grown *in vitro* consists of a few separate clumps of plasmosomin, each with peripheral aggregations of nucleolar chromatin (Fig. 21). That sarcoma cells may be indistinguishable from fibroblasts when both are grown under the same conditions *in vitro* is demonstrated by Fig. 40 and 41. The former is a photomicrograph of a 7-days-old primary culture of mouse-embryo-heart fibroblasts, and the latter of a 3-days-old culture of a sarcoma (Aa) which originated from the stroma of a spontaneous mammary carcinoma. Fig. 22 and 23 are drawings of the nucleolar system of two of the fibroblasts marked "×" in Fig. 40 ; and Fig. 24 and 25 of two of the sarcoma cells, similarly marked, in Fig. 41. Such close similarity in the morphology of fibroblasts and sarcoma cells is exceptional. Differences of the kind illustrated in Fig. 45 are commonest. Here are both fibroblasts and sarcoma cells, the latter marked "×". This photomicrograph was taken at the zone of contact of two cultures, one of mouse-embryo-heart fibroblasts, the other of sarcoma cells, from a tumour which had been induced by benzpyrene. Hypertrophy of the nucleolar system is a conspicuous feature of the sarcoma cells. This is also demonstrated by drawings in Fig. 26 and 27, and by the ultra-violet micrograph of Fig. 35. The binucleate cell shown in Fig. 26 was drawn from a culture of a sarcoma which had been induced by methylcholanthrene. The large nucleolus in the centre of each nucleus is seen to be connected by fine filaments to peripheral particles of chromatin. Fig. 27 depicts a cell from a Feulgen stained culture of a sarcoma (Rb) which originated by the sarcomatous transformation of the stroma of a mammary carcinoma. Only the plasmosomin and nucleolar chromatin are included in the drawing. Fig. 34 and 35 are ultra-violet micrographs of a cell of the same sarcoma, which was the most rapidly growing of the tumours which have been studied in the course of this work. Since all the Fig. 21 to 27 were drawn at the same magnification, it is obvious that the nucleolar system of malignant cells can undergo hypertrophy without a proportional increase in the volume of the nucleus.

### GENERAL CONSIDERATIONS.

To recapitulate, the distinctive features of nuclear structure are the chromosomes spread out over the inner surface of the nuclear membrane, and the nucleolar system consisting of nucleolar chromatin and plasmosome occupying the centre of the nucleus. Chromosomes with nucleolar organizers remain attached to the nucleoli, which are also connected with the periphery by fine hyaline strands. (Fig. 28 and 29).

The way in which the interphase nucleus develops from the telophase chromosomes has long been the subject of controversy among cytologists. Wilson (1925) in his review of the early literature recognised three principal types of nuclear

FIG. 21 to 27.—The nucleolar system of fibroblasts and sarcoma cells grown in tissue cultures. Nucleolar chromatin shown black ; plasmosomin stippled. Peripheral chromosomes omitted.

FIG. 21—Fibroblast from a 3-days-old culture (P).

FIG. 22, 23.—Fibroblasts from the 7-days-old culture photographed in Fig. 40. The cells shown in the drawings are marked " × " in Fig. 40 (P).

FIG. 24, 25.—Sarcoma cells (Aa) from the 3-days-old culture photographed in Fig. 41. The cells shown in the drawings are marked " × " in Fig. 41 (P).

FIG. 26.—Binucleate sarcoma cell from a methylcholanthrene induced tumour ($Cm_1$) showing fine filaments connecting the nucleolar system with the nuclear membrane (P).

FIG. 27.—Sarcoma cell from the most rapidly growing of the tumours (Rb) which were studied (P).

FIG. 28, 29.—Diagrammatic representation of nuclear structure, with surrounding mitochondria and cytoplasmic granules, as seen in surface view (Fig. 28), and in section (Fig. 29).

reconstruction. Of these the " simplest and rarest type " involved the formation of chromosomal vesicles, or karyomeres. This was "long ago described by Bütschli and Fol in the blastomeres of segmenting eggs and since observed in embryonic cells of many species. In this process each chromosome is converted into a small vesicle exactly like a minature nucleus, the whole group then fusing together progressively so as to form first an irregular, chambered structure and finally a single nucleus. From the outer wall of this, apparently, arises the nuclear membrane, while the inner walls of the vesicles break down irregularly to form the nuclear network". More recently Lewis (1947) has suggested a similar mode of nuclear reconstruction in mammalian cells in tissue cultures. He considers that the interphase nucleus " probably consists of closely packed, firmly adherent, swollen chromosomes (chromosomal vesicles or karyomeres)." This condition is brought about by the telophase chromosomes swelling to form vesicles which adhere to one another. Each vesicle retains its identity throughout the interphase. " During prophase each chromosome sinks into a dense gel which is separated from its neighbours by the fluid which collects between them." Lewis (1947) points out that amitosis and nuclear fragmentation would be readily explicable as resulting from the loss of adhesion between chromosomal vesicles.

Since nuclear formation by the fusion of chromosomal vesicles is said to be specially characteristic of embryonic cells, it would not be surprising to find the same process occurring in malignant cells which they resemble in many respects. Karyomeres are often clearly distinguishable in dividing cancer cells. Attention has already been directed to the example in the middle of Fig. 38. Other instances following irradiation are depicted in Fig. 11 and 12. In these cases they are particularly conspicuous because of their delayed fusion. The nuclear structure which has been postulated, and is shown diagrammatically in Fig. 28 and 29 could originate from the cohesion of karyomeres, formed, not as Lewis (1947) suggested, by the swelling of chromosomes, but by their spreading out over the surface of vesicles. After coalescence of the latter, the chromosomes would adhere to the internal surface of the external walls of the vesicles, which form the nuclear membrane. Instead of the inner walls of the vesicles breaking down irregularly to form a nuclear network as Wilson (1925) suggested, they would constitute the strands which extend between the nucleoli and the cell membrane. To bring about such an arrangement it would be necessary to postulate the existence of some mutually repellant force between the chromosomes and the nucleolus at this stage, so that the latter was forced into the centre of the coherent vesicles.

From a cursory examination which has been made of different tissues of a number of mammals, including man, it appears that their nuclear structure is essentially the same as that which has been described, but no comparable study has been made of the nuclei of lower animals, or plants. Apparently there is a fundamental difference between the nuclear structure of the higher animals and the higher plants. According to the recent work of Chayen, Davies and Miles (1953) the interphase nucleus of plants (*Vicia, Allium, Zea, Tradescantia*) consists of a peripheral zone containing the chromosomes, a middle zone with a protein content, the width of which varies with the state of mitotic activity, and an inner zone occupied by the nucleoli. Each of the latter is composed of a central core of protein material surrounded by a substance, which probably contains nucleoprotein. There is nothing comparable to the clearly defined differentiation between the nucleolar chromatin and plasmosomin of the mammalian nucleolar system. " When two nucleoli are present the middle zone is divided in two by peripheral zone material, so that each nucleolus lies in a pouch filled with middle zone protein and lined by the peripheral zone." A corresponding structure has not been observed in mammalian cells with more than one nucleolus (Fig. 34–37), but a feature common to both is the occurrence of a fine chromosomal strand connecting the nucleolar organizer with the periphery of the nucleus.

Any discussion of the functional significance of the nuclear organization of the animal cell, which has been postulated, would be incomplete unless it took into consideration the structure of the surrounding cytoplasm. In rapidly growing tumour cells the nucleus is closely invested by mitochondria. Its structure and spatial relationship with the surrounding mitochondria are depicted diagrammatically in Fig. 28 and 29. The former attempts to portray the nucleus as it would appear in surface view. The latter shows the interior of the nucleus as it might look if sliced through its middle. The most striking morphological feature is the manner in which most of the chromosomes are spread out over a spherical area, thereby bringing about maximum exposure to the cytoplasm on the outside, and to substances emitted from the nucleolus on the inside. Some of the mitochondria and other granules are applied to the surface of the nucleus, and only the thickness of its membrane separates them from the chromosomes. Clearly, substances which penetrate the plasma membrane must pass through the cordon of mitochondria before reaching the nucleus, and between the chromosomes if they are to get to the nucleolar system.

In pioneer studies on living cells in tissue cultures, Lewis and Lewis (1915) followed the movements of mitochondria backwards and forwards between the nucleus and the cell periphery, and recently Pomerat (1953) has observed rotary movements of the nucleus in epithelial cells growing *in vitro*. Reasons have already been adduced for believing that the mitochondria are involved in protein synthesis (Ludford, Smiles and Welch, 1948*a*, 1948*b*.), and attention has been directed to the correlation between hypertrophy of the nucleolar system, proliferation of mitochondria and increased nucleotide content of the cytoplasm (Ludford, 1951). Such observations as these tend to emphasise that the cell as a whole is a unified functional system. It is reasonable to suppose that the performance of its fundamental vital processes necessitates intimate correlation of the functional activities of all its organellae.

Recently Brenner (1953) has deduced from the appearance presented by the nucleus in phase-contrast photomicrographs and ultra-violet micrographs (Ludford and Smiles, 1950*a*, 1950*b*.), and from the results of ultra-centrifugation experiments carried out by himself and others (Beams, 1948 ; Claude, 1943) that segments of chromosomes are attached to the inner surface of the nuclear membrane. " These segments maintain their continuity with the remaining parts of the chromosomes which, as uncoiled threads, occupy the inside of the interphase nucleus." On the basis of Caspersson's (1950) hypothesis that heterochromatic regions of the chromosomes control protein synthesis Brenner (1953) has proposed that " the nuclear membrane heterochromatin with its specific genes controls the synthesis of equally specific microsomes, each desoxyribonucleic acid directing the synthesis of a characteristic type of ribonucleoprotein".

" Protein synthesis is a two phase reaction", according to Haurowitz and Crampton (1952). In its first phase, an expanded peptide film acts as a template on which a layer of amino acids is absorbed. A non-specific enzyme is responsible for peptide bond formation between the absorbed amino acids, so that a second identical peptide film is formed. The peptide film is maintained in the expanded state, its only efficient form, by combining with nucleic acids to form a nucleoprotein. In the second phase of protein synthesis, the peptide chain is folded to form a three-dimensional globular molecule. Haurowitz and Crampton (1952) suggest that the highly specific peptide chains are probably first formed in the nucleus, and that they pass out into the cytoplasm where they are specifically folded. Since antigens are deposited mainly in cytoplasmic granules in liver cells it is concluded that the folding of the peptide chains takes place in these granules. As Haurowitz and Crampton (1952) admit, their evidence that the first phase occurs inside the nucleus is equivocal, while certain of their experimental results indicate that it takes place in the cytoplasmic granules. Mudd (1952) has pointed out that it is logical to think that synthetic reactions occur in the neighbourhood of mitochondria, since the latter are " the chief source of energy supply in the cell, of the efficient aerobic type of respiration which yields energy which is coupled with energy-requiring reactions of synthesis". Morphological studies on mitochondria in cells under various physiological and pathological conditions strongly support this view (Bourne, 1950, 1951 ; Ludford, 1951*a*).

These conflicting ideas which emphasise on the one hand, the activities of the cytoplasmic organellae, and on the other, the role of the nucleus, are not mutually contradictory. While there is considerable evidence to support the contention that mitochondria are concerned with cell respiration, there are equally good reasons for believing that genes either elaborate enzymes or control their activities by producing specific activators or inhibitors. It is reasonable to assume that the close spatial relationship between mitochondria and chromosomes (Fig. 28 and 29) is the morphological expression of an intimate functional

---

### DESCRIPTION OF PLATES

Fig. 30, 31.—Ultra-violet micrographs (2750 Å) of nuclei of a mammary carcinoma (Af).
Fig. 30.—Isolated coagulated nuclei in saline.
Fig. 31.—Similar nuclei after smearing.
Fig. 32 to 37.—Ultra-violet micrographs of nuclei of living carcinoma and sarcoma cells (2750 Å).
Fig. 32.—Part of the nucleus of a mammary carcinoma cell (Af) with the surface of the nucleus in focus.
Fig. 33.—Part of the nucleus of another similar cell with the nucleolar system in focus.
Fig. 34, 35.—Two optical sections through the same sarcoma cell (Rb). In Fig. 34 the peripheral granules (chromosomes) are seen; and in Fig. 35 the nucleolar system occupies the centre of the nucleus.
Fig. 36.—Prophase nucleus of a sarcoma cell (Rb) showing the peripheral chromosomes.
Fig. 37.—Similar cell with the internal nucleolar system in focus.
Fig. 38, 39.—Malignant mammary gland cells and their non-malignant prototypes in tissue cultures.
Fig. 38.—Three-days-old culture of mammary carcinoma (Ad).
Fig. 39.—Five-days-old culture of mammary gland from a mouse of a low-breast-cancer strain.
Fig. 40, 41.—Sarcoma cells and their non-malignant prototypes in tissue cultures.
Fig. 40.—Seven-days-old culture of fibroblasts.
Fig. 41.—Three-days-old culture of sarcoma (Aa).
Fig. 42 to 45.—Fibroblasts and sarcoma cells in tissue cultures.
Fig. 42, 43.—Fibroblast nuclei showing peripheral granules representing chromosomes and the nucleolar system so flattened as to be almost in the same plane.
Fig. 44.—Early prophase nucleus of a fibroblast containing chromosomes with conspicuous end knobs.
Fig. 45.—Zone of contact of cultures of fibroblasts and sarcoma cells (Bb3). The malignant cells marked " × ".

30

31

Ludford.

32
33
34
35
36
37

38

39

Ludford.

40

41

Ludford.

42 43 44

45

Ludford.

relationship. The peptide film which Haurowitz and Crampton (1952) suggest acts as a template in protein synthesis might be localised on the surface of the mitochondria. Occasionally fibroblasts migrating from explants in tissue cultures leave behind them small pieces of cytoplasm which contain mitochondria and other granules. Growth of such enucleate cytoplasm has not been observed by the writer. One possible reason for this is that the function of mitochondria is controlled by substances produced by the chromosomes. Certain observations also suggest that the latter are dependent upon substances supplied by the nucleolar system.

In conclusion, it should be pointed out that cells may undergo malignant transformation without any alteration occurring in their nuclear structure that can be detected by the present methods of microscopy. This is understandable if as has been suggested (Ludford, 1952) the cytological basis of malignancy is an imbalance in the genes brought about by processes which result in (1) the loss of some chromosomes, or parts of chromosomes, and (2) the intranuclear duplication of others. This implies that with improvements in microscopical technique it should be possible to distinguish differences between the chromosomes which are spread out on the inner surface of the nuclear membranes of malignant cells and their non-malignant prototypes. Our present microscopical methods are inadequate for obtaining really satisfactory pictures of the surface of the nucleus. In fixed and stained preparations chromosomes look like strings of irregular-shaped beads, strung on invisible short wavy threads (Fig. 1 to 5, and 42 and 43). Some ultra-violet micrographs exhibit the same arrangement (Fig. 32 and 34). Others suggest that the granular material is adherent to the surface of fine hyaline threads or tubules. Instead of tubules there may be rows of minute droplets the coalescence of which could be responsible for the formation of filaments. Evidence supporting such a probability is afforded by ultra-violet micrographs of smears of coagulated nuclei (Fig. 31). Unfortunately the methods employed in this work do not give high enough resolution to yield sufficiently convincing results for making a proper study of these intricate structures. Clearly, progress in this direction is dependent upon the devising of new techniques.

The nuclear structure of the cells of most malignant growths exhibit a variety of abnormalities. Hypertrophy of the nucleolar system and an increase in chromatin content are of common occurrence. According to Caspersson and Santesson (1942) the heterochromatin system of malignant cells is stimulated to abnormal activity. This leads to disturbances in the formation of cytoplasmic proteins and in the reproduction of gene protein, which result in abnormal growth. It would be more compatible with the results of the present study if hypertrophy of the nucleolar system and a hyperchromatinic condition of the nucleus were the morphological expression of an increased growth rate rather than being indicative of malignant growth.

It has not been possible to identify the nucleolar organizer on the chromosomes of the cells examined during the course of this work. So it remains to be determined whether increase in size of the nucleolar system is the result of duplication of chromosomes bearing nucleolar organizers. There is, however, indirect evidence that this may be the case (Biesele, Poyner and Painter, 1942).

The studies with ultra-violet microscopy described in this paper were carried out in the National Institute for Medical Research and constitute part of a wider research on the application of modern methods of microscopy to the study of living malignant cells (Ludford, Smiles and Welch, 1948*a*, 1948b; Ludford and Smiles, 1950*a*, 1950*b*).

SUMMARY.

1. The distinctive features of the nucleus of the mammalian cell are—

(i) most of the chromosomes are spread out over the inner surface of the nuclear membrane.
(ii) the nucleolar system occupies the middle part of the nucleus. In its simplest form it consists of a single plasmosome bearing peripheral aggregations of nucleolar chromatin. In its most complex form in tumour cells it consists of numerous smaller bodies comprised of the same two constituents.
(iii) chromosomes, presumably bearing nucleolar organizers, remain attached to nucleoli, and some at least extend from nucleoli to the nuclear periphery, as do also other delicate strands, probably composed of the same material as that which forms the nuclear membrane.

This interpretation of nuclear structure is depicted diagrammatically in Fig. 28 and 29.

2. Changes observed in the nucleolar system are explicable on the assumption that either nucleolar chromatin is formed by the plasmosomes, or that both nucleolar constituents are synthesised from the same simple precursors, and that there are alternative pathways of metabolism leading to the formation of one or the other substance.

3. In rapidly growing cells the proximity of mitochondria to the chromosomes immediately under the nuclear membrane implies functional interrelationship. It is suggested that protein synthesis occurs at the surface of the mitochondria and is controlled by substances emitted by the chromosomes, and that the nucleolar system supplies substances needed for the activity of the interphase chromosomes. Deoxyribonucleoproteins from the nucleolar chromatin and ribonucleoproteins from the plasmosome contribute at prophase to the formation of the mitotic chromosomes.

4. Cells may undergo malignant transformation without alteration of nuclear structure that can be detected by the present techniques of microscopy. Nevertheless the nuclei of most malignant cells exhibit hypertrophy of the nucleolar system, accompanied by an increase in extra-nucleolar chromatin (deoxyribonucleoprotein.)

### REFERENCES.

BEAMS, H. W.—(1948) *J. Morph.*, **83**, 87.
BENSLEY, R. R.—(1938) *Anat. Rec.*, **72**, 351.
BIESELE, J. J., POYNER, H., AND PAINTER, T. S.—(1942) 'Nuclear Phenomena in Mouse Cancers.' University of Texas Publications, No. 4243.
BITTNER, J. J.—(1951) *Trans. Stud. Coll. Physic. Philadelphia*, **9**, 129.
BORYSKO, E., AND BANG, F. B.—(1952). *Johns Hopk. Hosp. Bull.*, **89**, 468.
BOURNE, G. H.—(1950) *J. R. micr. Soc.*, **70**, 367.—(1951) 'Mitochondria and the Golgi Complex. Cytology and Cell Physiology.' Oxford (Clarendon Press), p. 232.
BRENNER, S.—(1953) *Exp. Cell Res.*, **5**, 257.

CASPERSSON, T. O.—(1947) 'The Relations between Nucleic Acid and Protein Synthesis.' *Symp. Soc. exp. Biol.*, No. 1. p. 127.—(1950) 'Cell Growth and Cell Function.' New York (W. W. Norton & Co.).

*Idem* AND SANTESSON, L.—(1942) *Acta Radiol.*, Stockh., Suppl. 46.

CHAMBERS, R.—(1924) 'The Physical Structure of Protoplasm as determined by Microdissection and Injection. General Cytology,' edited by E. V. Cowdry. (University of Chicago Press), p. 235.

*Idem* AND LUDFORD, R. J.—(1932) *Arch. exp. Zellforsch*, **12**, 555.

CHAYEN, J., DAVIES, H. G. AND MILES, U. J.—(1953) *Proc. Roy. Soc.*, B., 141, 190.

*Idem* AND NORRIS, K. P.—(1953) *Nature*, **171**, 472.

CLAUDE, A.—(1943) 'Distribution of Nucleic Acids in the Cell and the Morphological Constitution of Cytoplasm. Frontiers in Cytochemistry.' *Biol. Symp.* **10**, 111. Edited by N. L. Hoerr. (Jaques Cattel Press, Lancaster Pa).

*Idem* AND POTTER, J. S.—(1943) *J. exp. Med.*, **79**, 345.

GATES, R. R.—(1942) *Bot. Rev.*, **8**, 337.

HAUROWITZ, F., AND CRAMPTON, C. F.—(1952) *Exp. Cell Res.*, Suppl. 2, p. 45.

HOERR, N. L.—(1943) 'Methods of Isolation of Morphological Constituents of the Liver Cell.' *Biol. Symp.*, **10**, 185.

KIRSCHBAUM, A., AND BITTNER, J. J.—(1945) *Proc. Soc. exp. Biol., N. Y.*, **58**, 18.

LAMB, W. G. P.—(1950) *Exp. Cell Res.*, **1**, 571.

LEWIS, W. H.—(1947) *Anat. Rec.*, **97**, 433.

*Idem* AND LEWIS, M. R.—(1915) *Amer J. Anat.*, **17**, 339.

LUDFORD, R. J.—(1924) *Proc. Roy. Soc.*, B, **97**, 50.—(1930) *Sci. Rep. Imp. Cancer Res. Fd.*, **9**, 149.—(1932*a*) *Ibid.*, **10**, 125.—(1932*b*) *Ibid.*, **10**, 169.—(1933) *Arch. exp. Zellforsch.*, **14**, 42.—(1934) *Sci. Rep. Imp. Cancer Res. Fd.*, **11**, 147.—(1935) *Protoplasma*, **23**, 180.—(1951*a*) 'Pathological Aspects of Cytology. Cytology and Cell Physiology,' edited by G. H. Bourne. Oxford (Clarendon Press), p. 373.—(1951) *Ann. Rep. Brit. Emp. Cancer Campgn.* **29**, 306.—(1952) *Ibid*, **30**, 361.

*Idem* AND SMILES, J.—(1950*a*) *J. R. micr. Soc.*, **70**, 186.—(1950*b*) *Ibid*, **70**, 194.

*Iidem* AND WELCH, F. V.—(1948*a*) *Nature*, **162**, 650.—(1948*b*) *J. R. micr. Soc.*, **68**, 1.

MIRSKY, A. E., AND POLLISTER, A. W.—(1943) "Fibrous Nucleoproteins of Chromatin." *Biol. Symp.*, **10**, 247.

MOYER, L. S. (1942) 'Proteins and Protoplasmic Structure. The Structure of Protoplasm,' edited by W. Seifriz. (Iowa State College Press), p. 23.

MUDD, S.—(1952) *Exp. Cell Res.*, Suppl. 2, p. 56.

PAGE, R. C., REGAN, J. F., MACCARTY, W.C.—(1938) *Amer J. Cancer*, **32**, 383.

POLLISTER, A. W.—(1952) *Exp. Cell. Res.*, Suppl. 2, p. 59.

POMERAT, C. M.—(1953) *Ibid.* **5**, 191.

SPARROW, A. H., AND HAMMOND, M. R.—(1947) *Amer. J. Bot.*, **34**, 439.

WILSON, E. B.—(1925) 'The Cell in Development and Heredity.' New York. (MacMillan Co.).


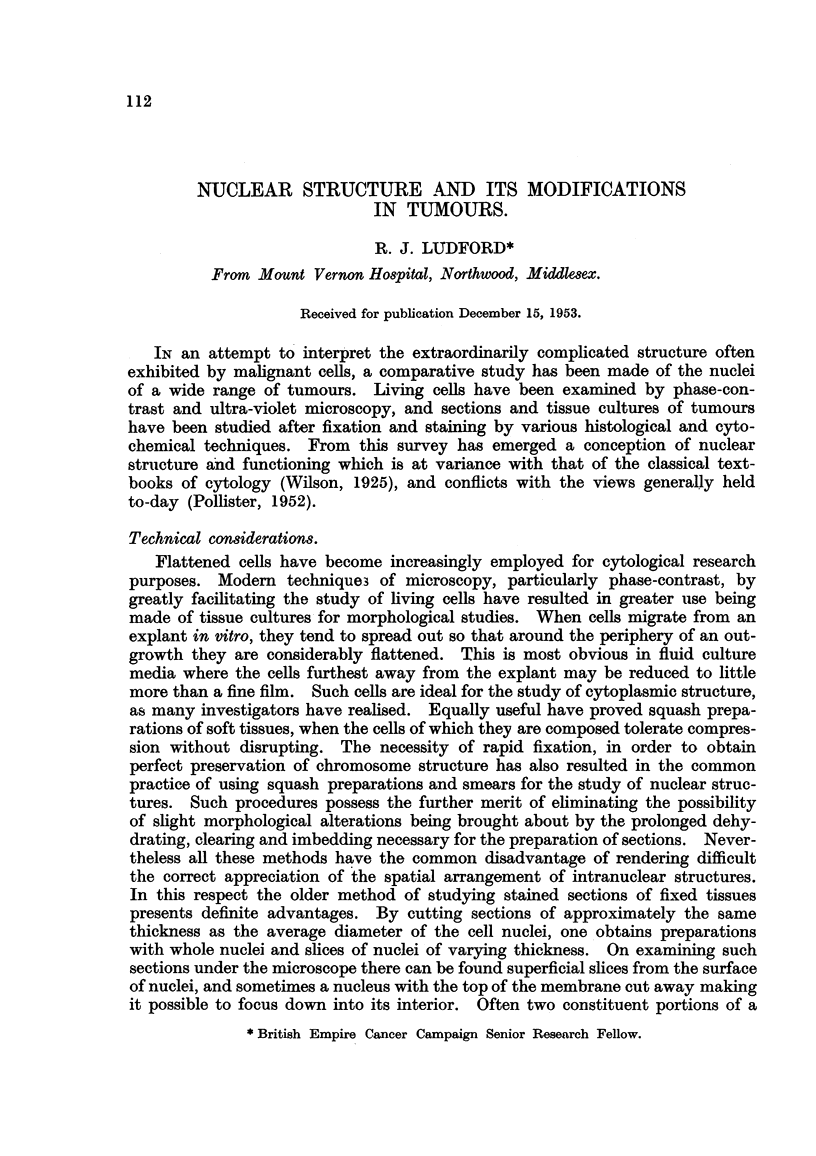

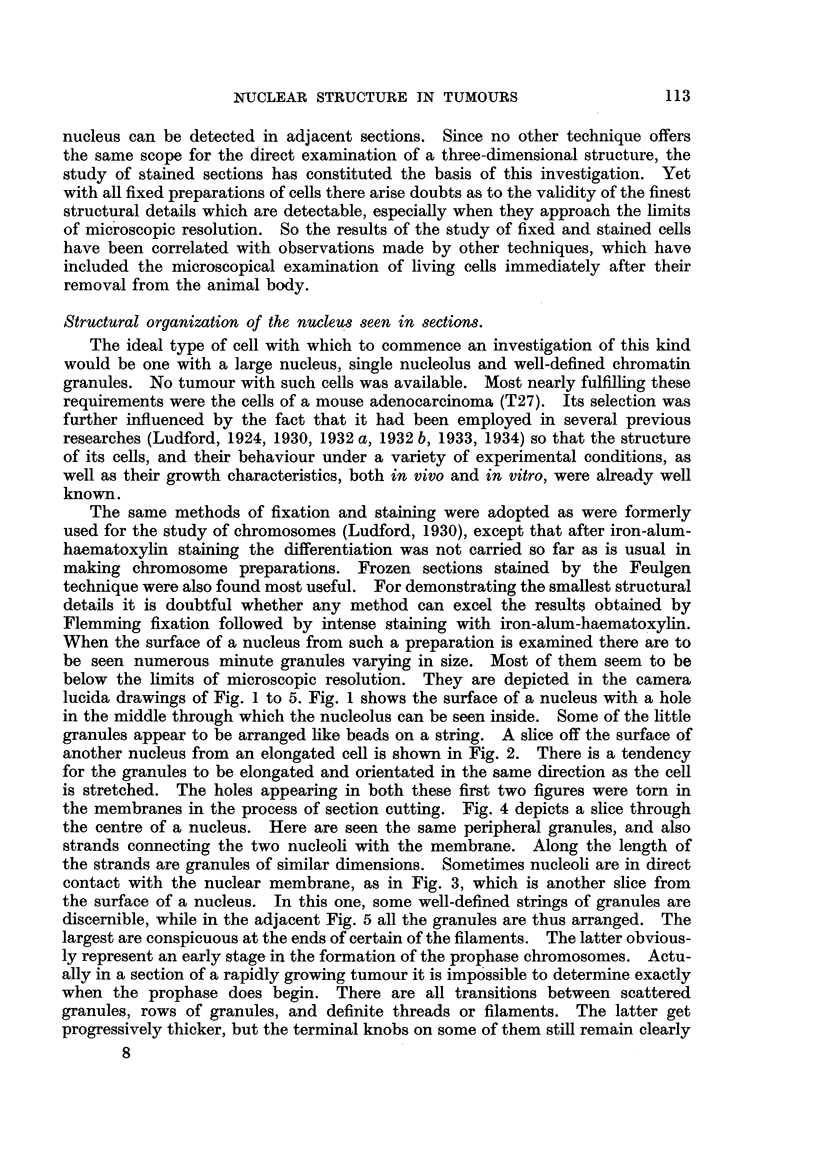

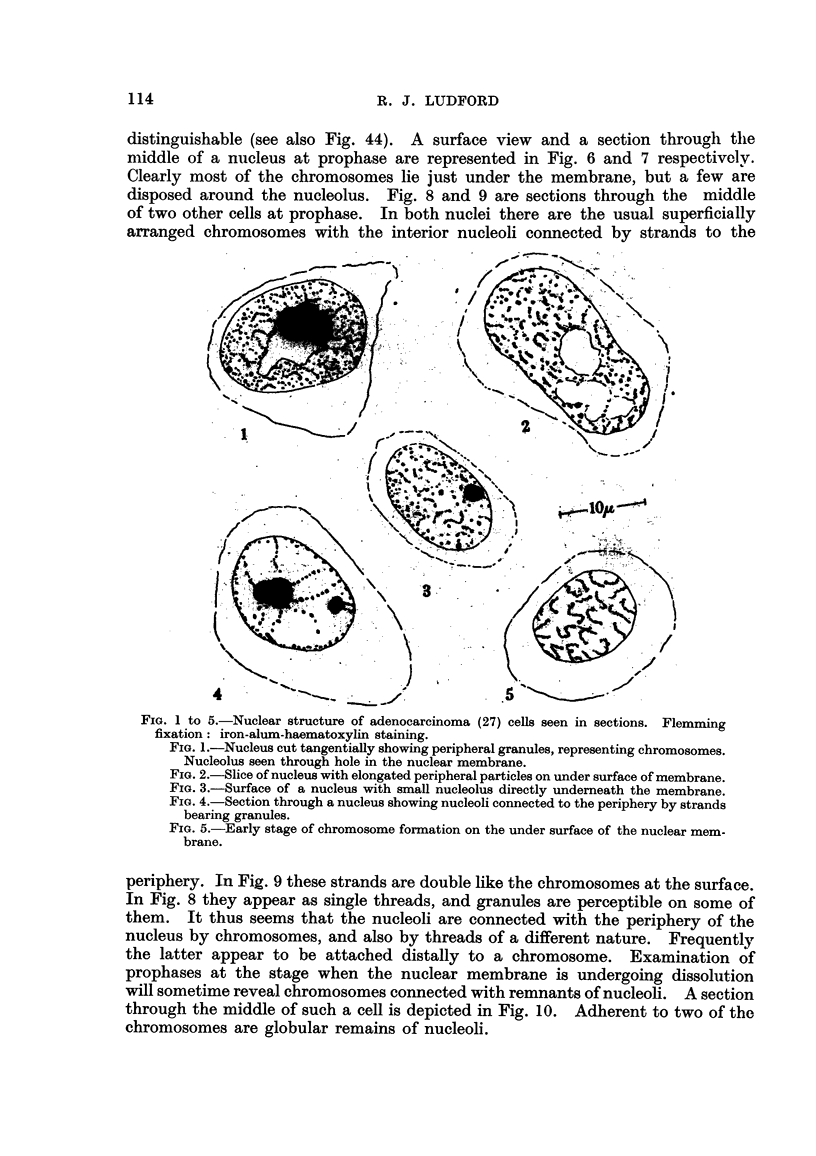

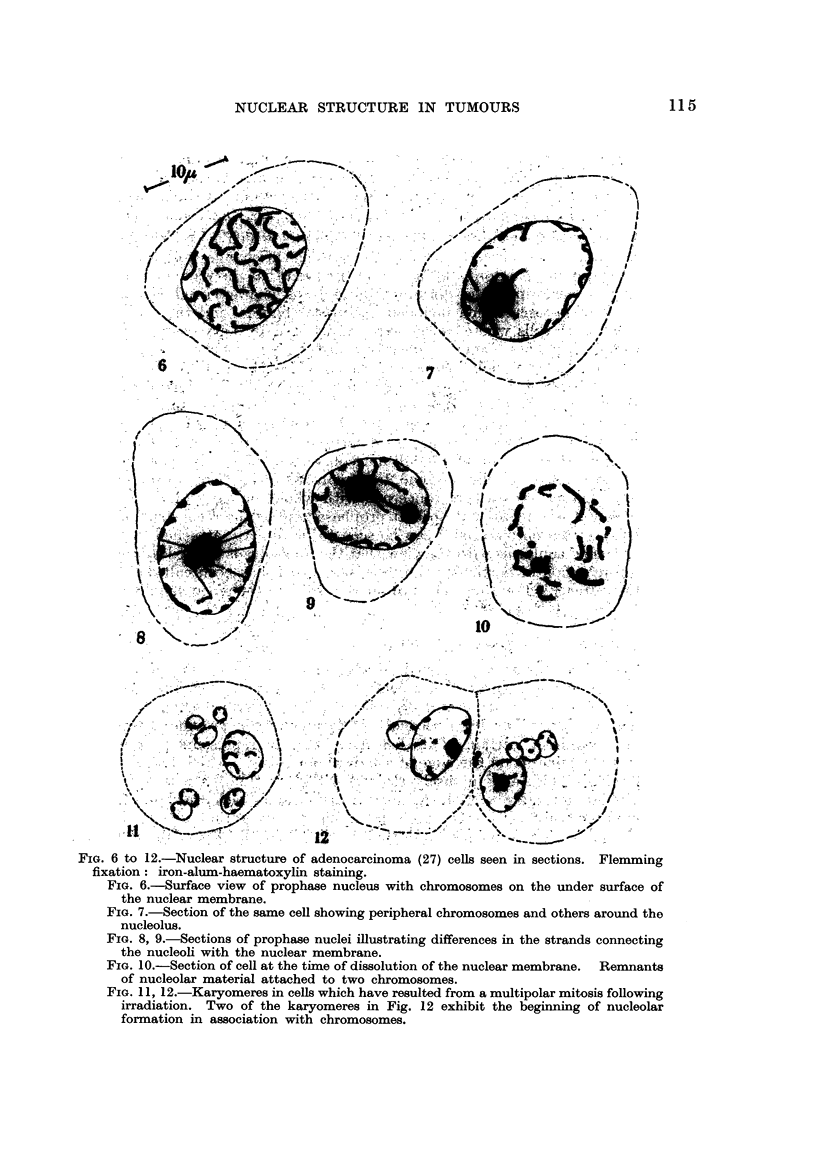

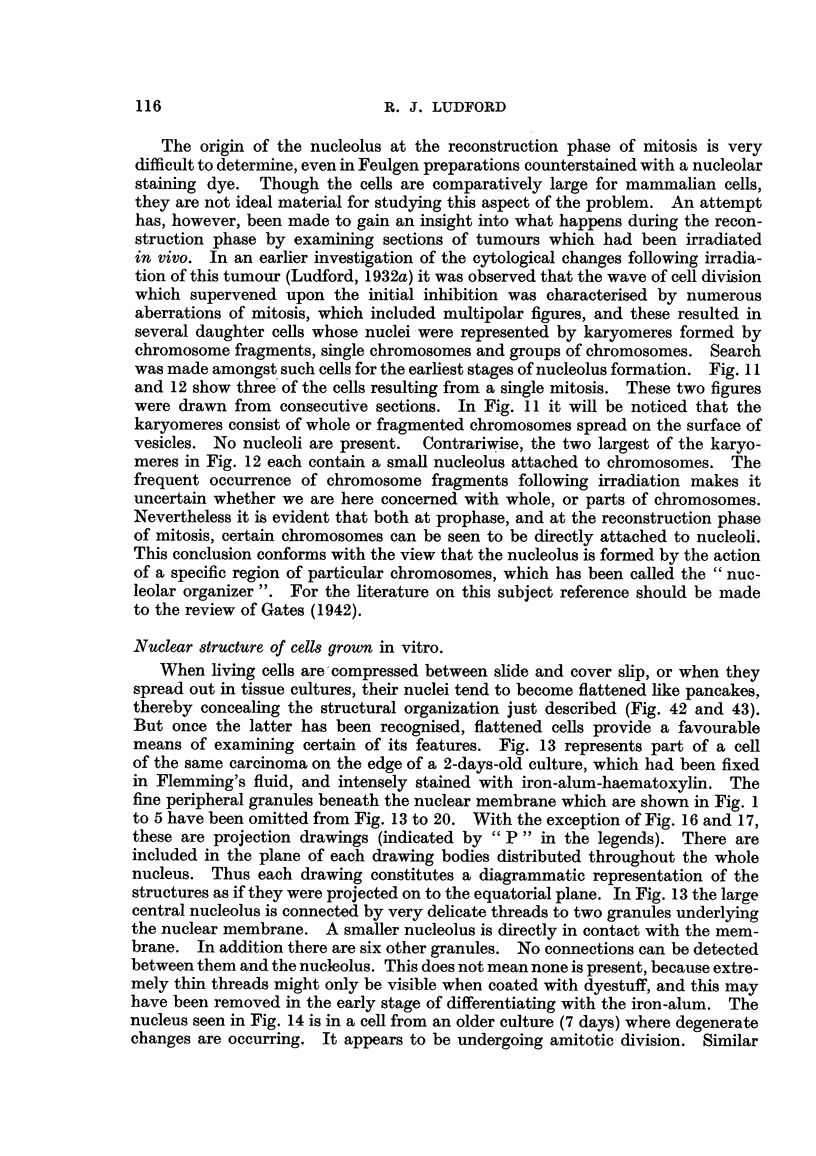

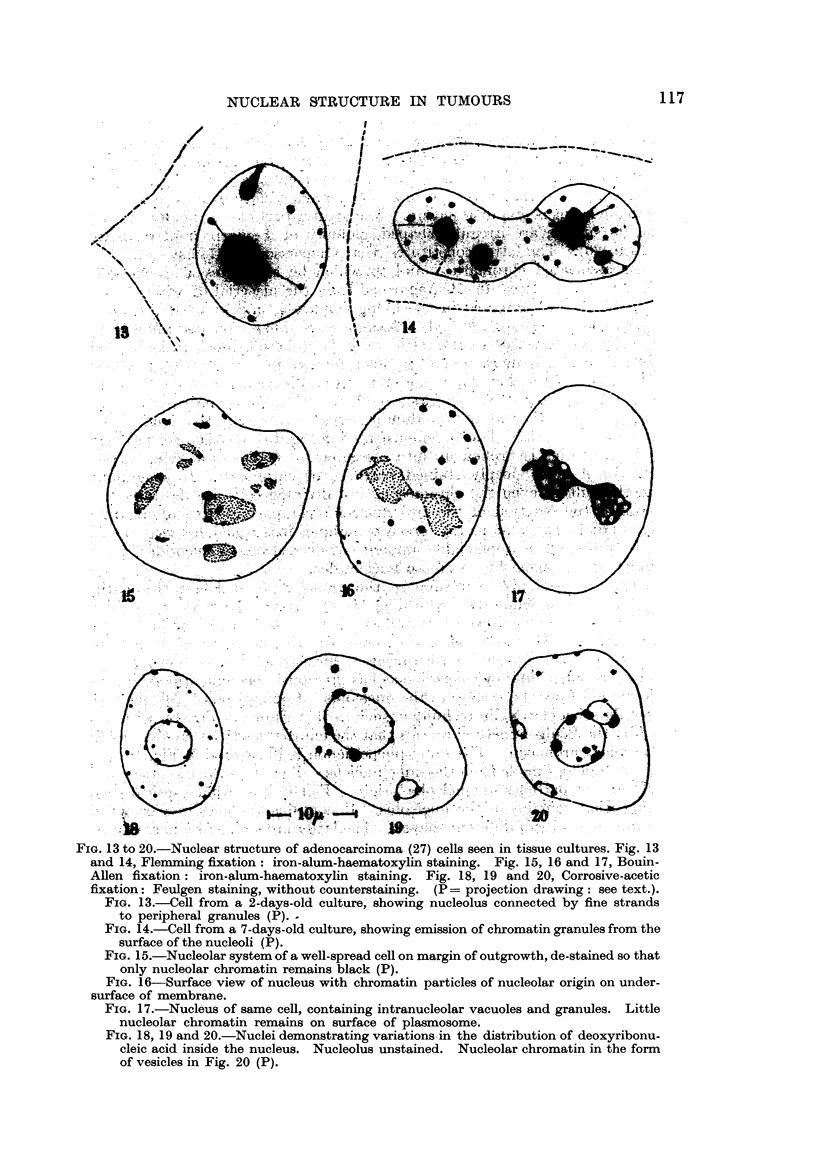

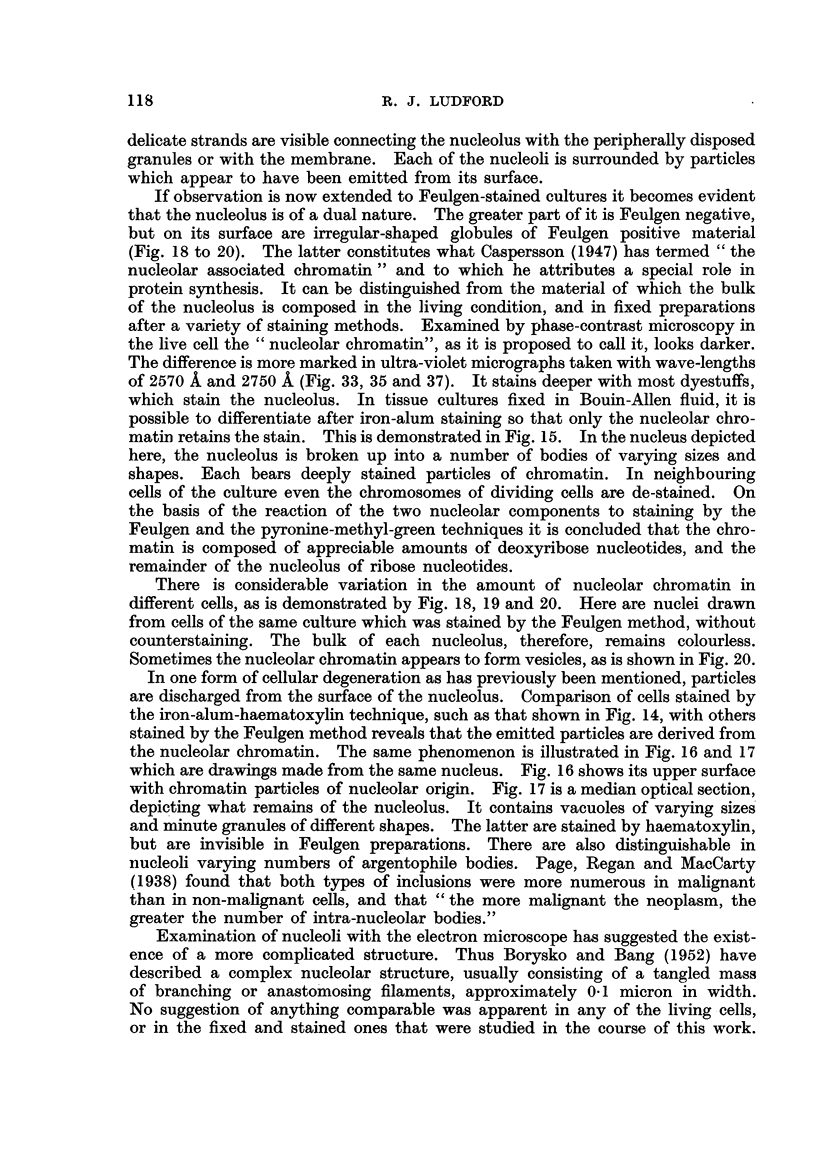

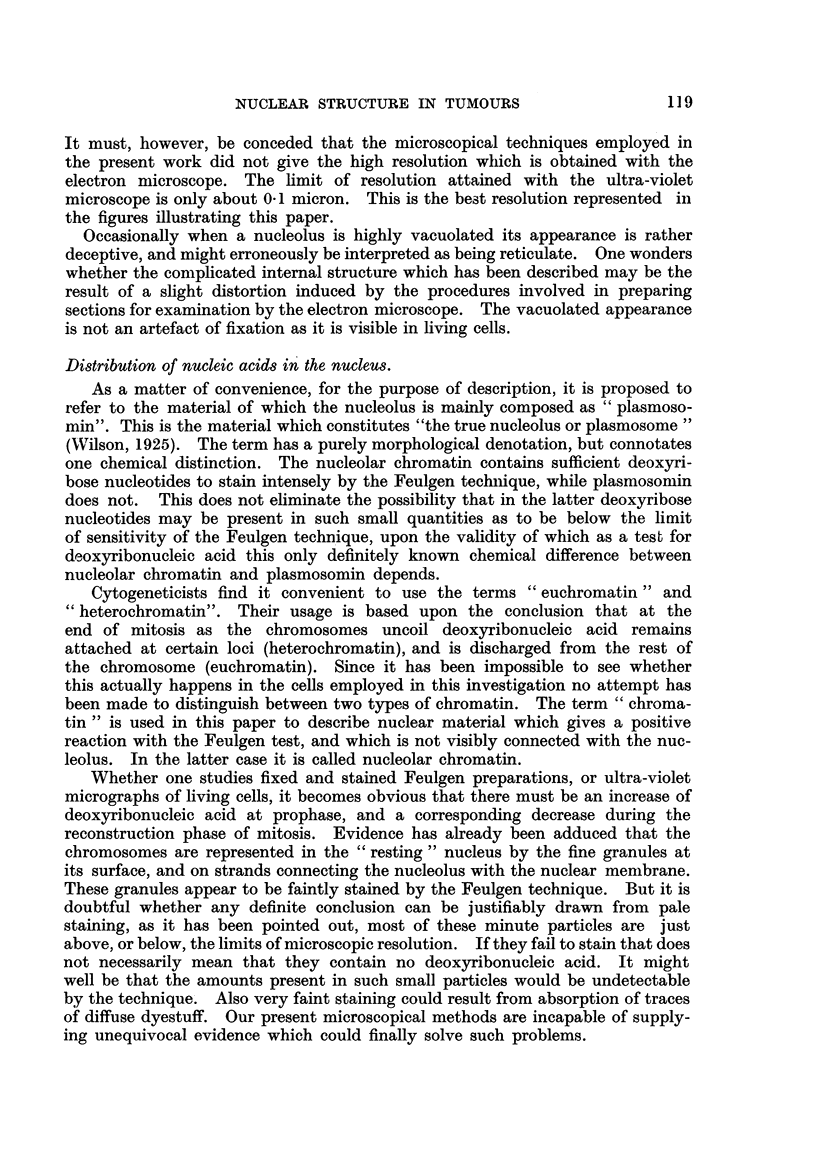

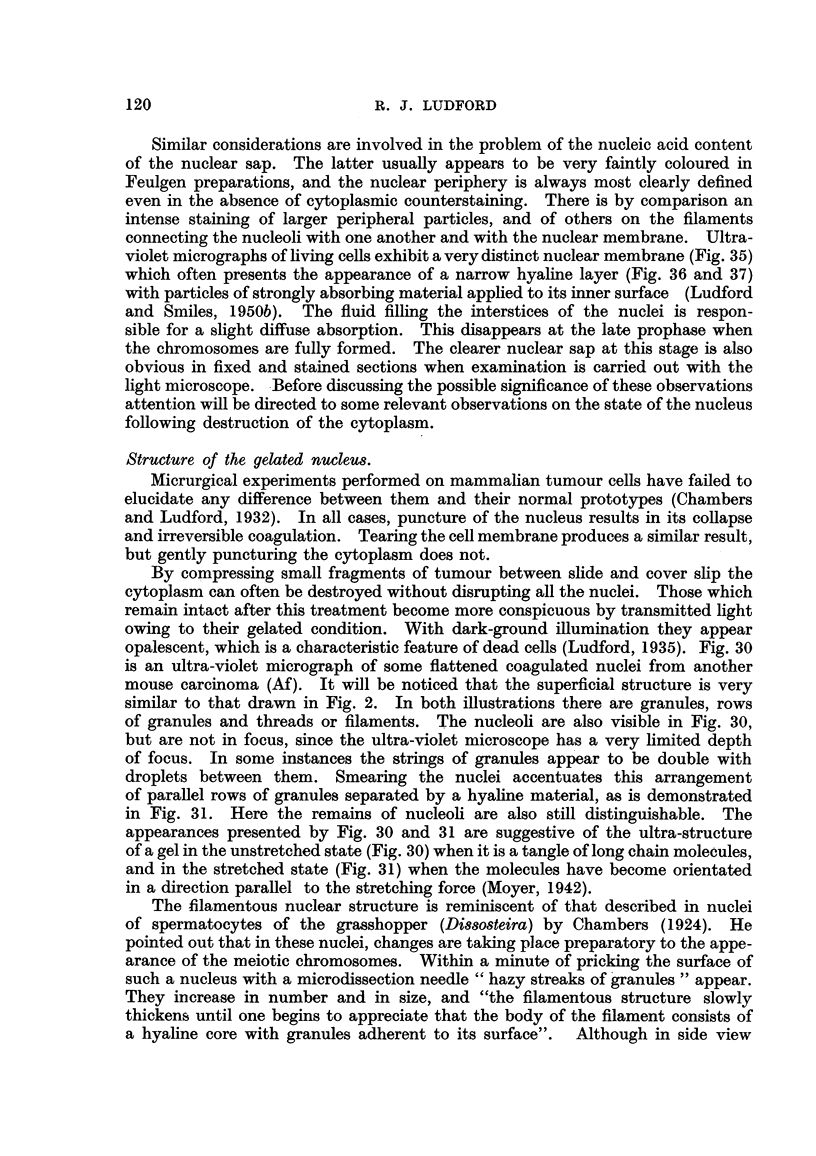

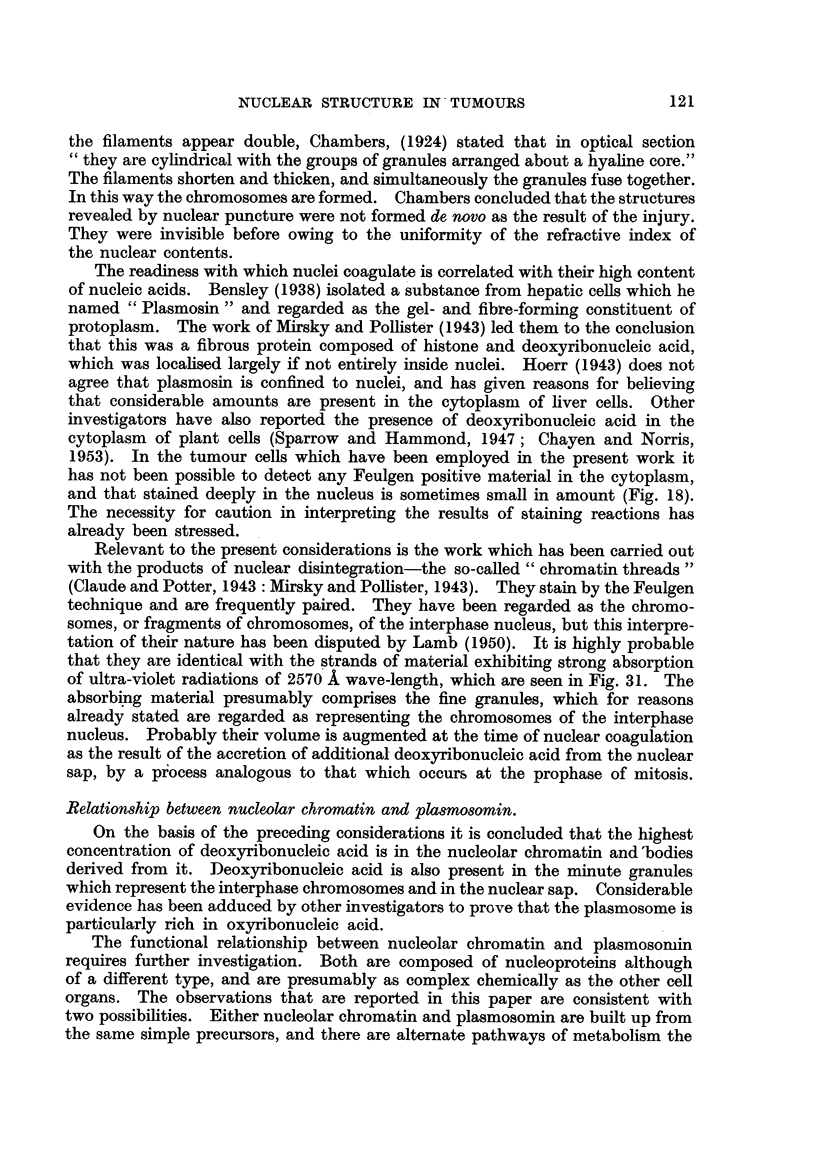

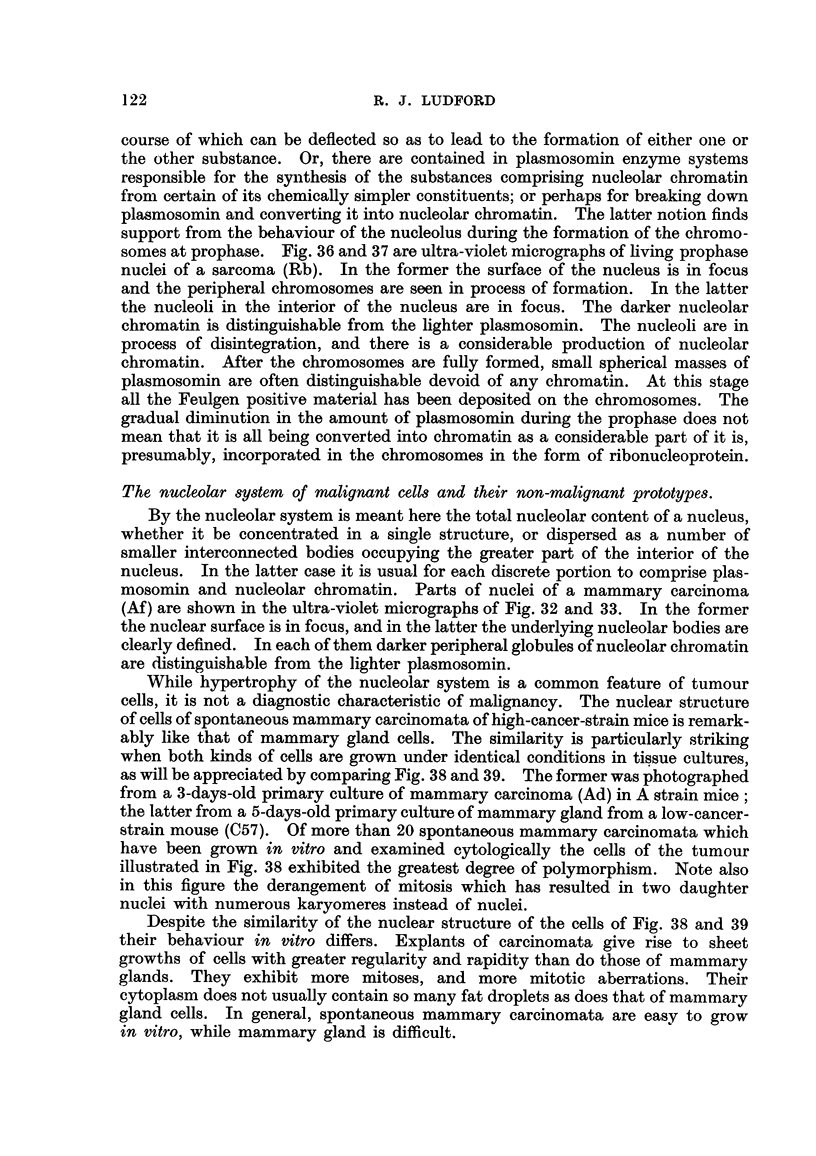

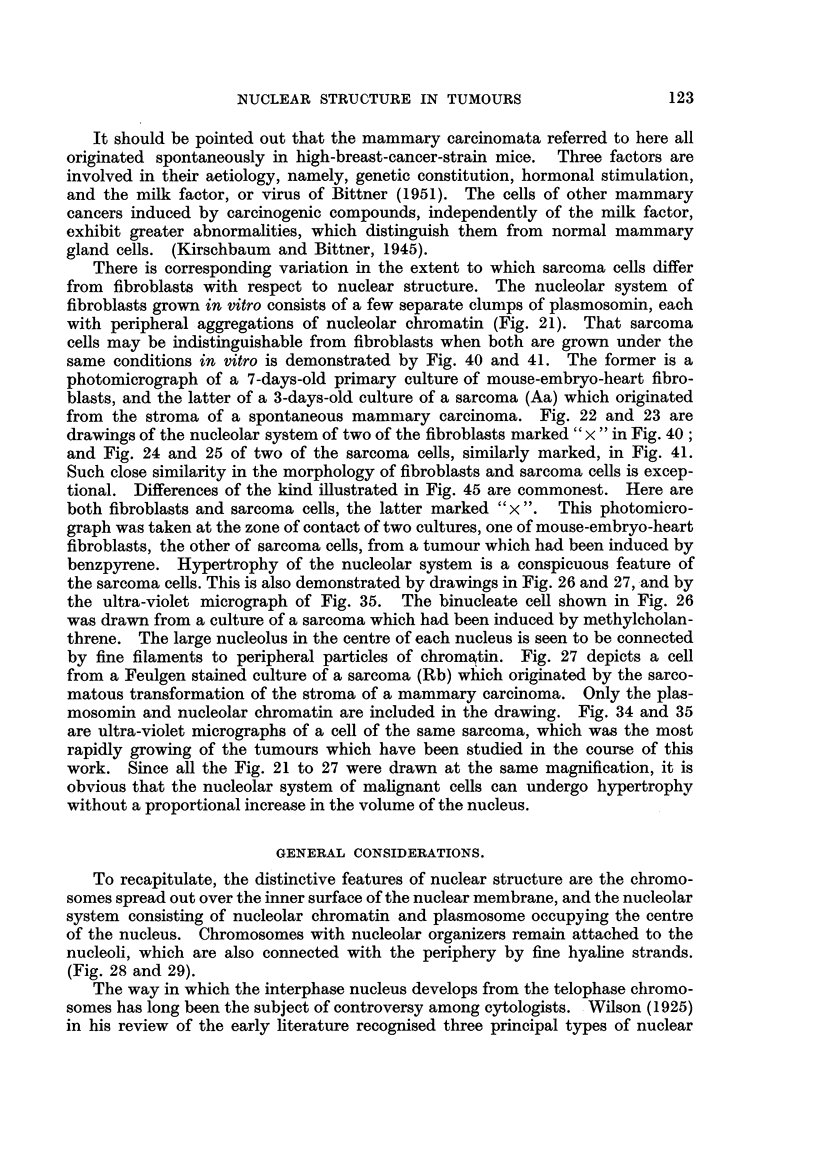

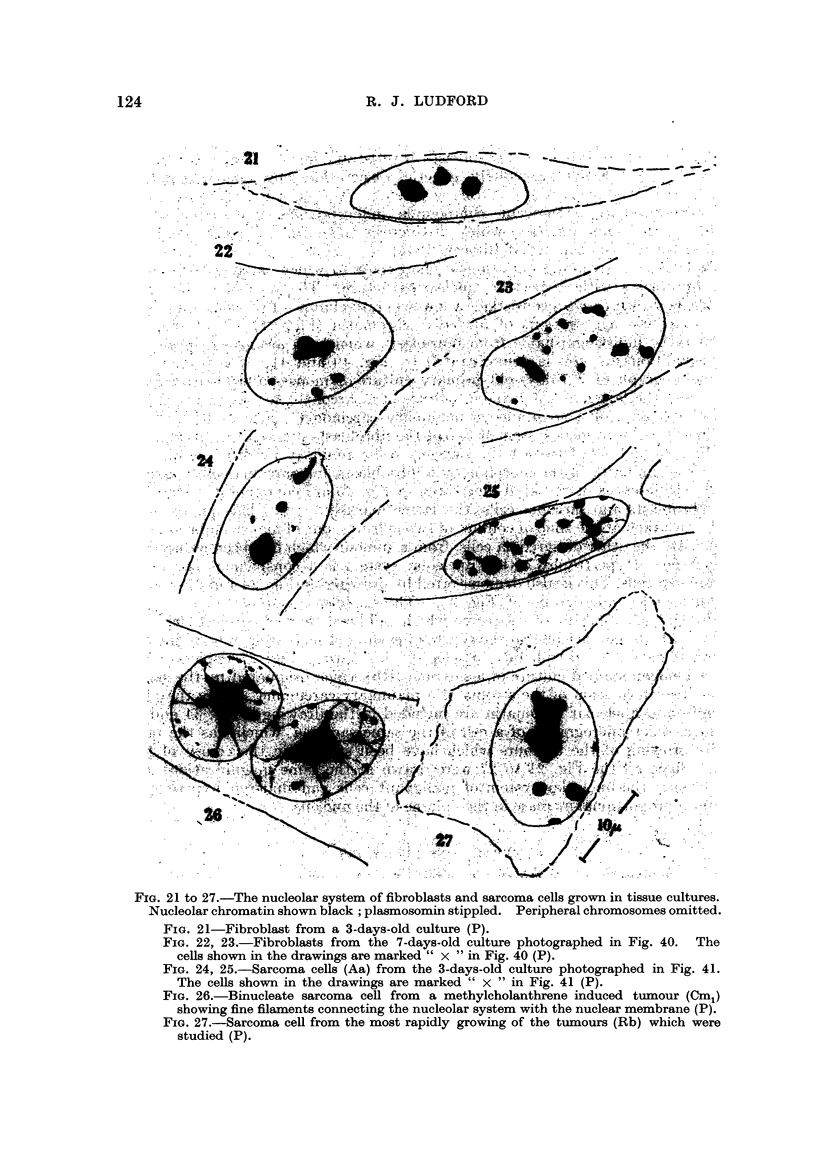

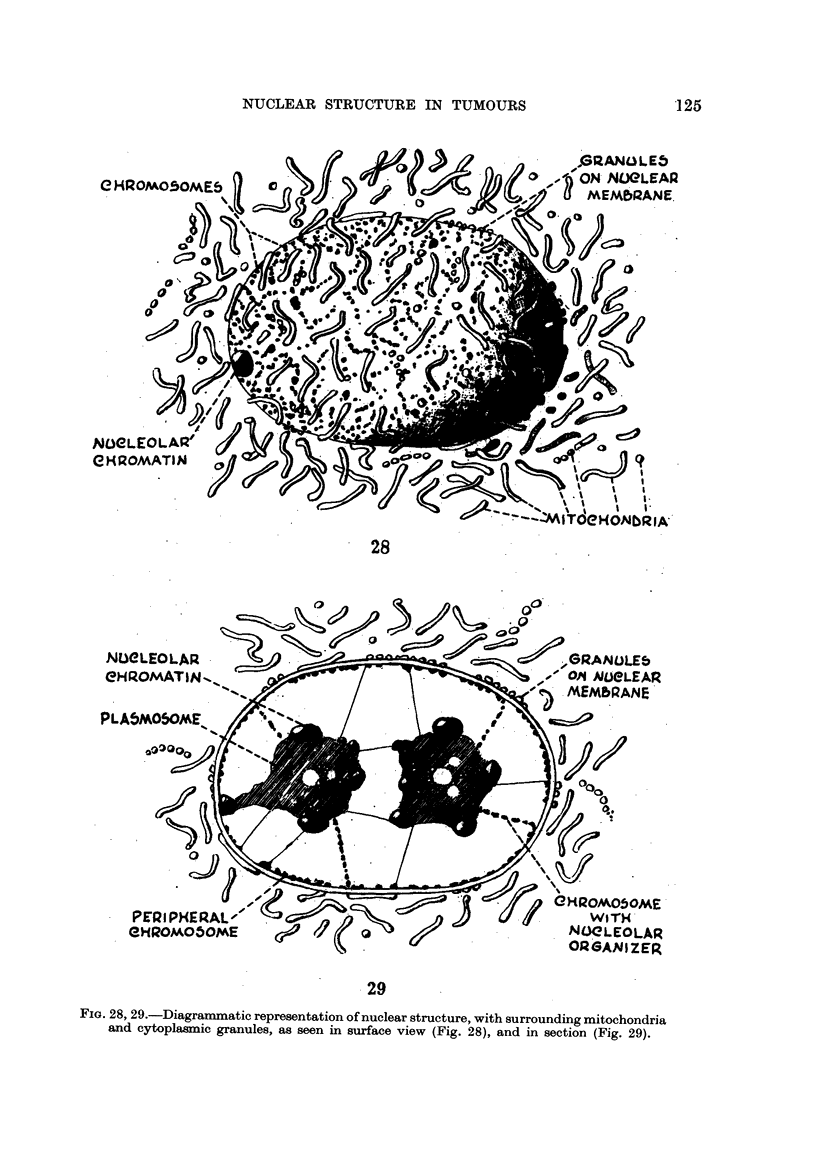

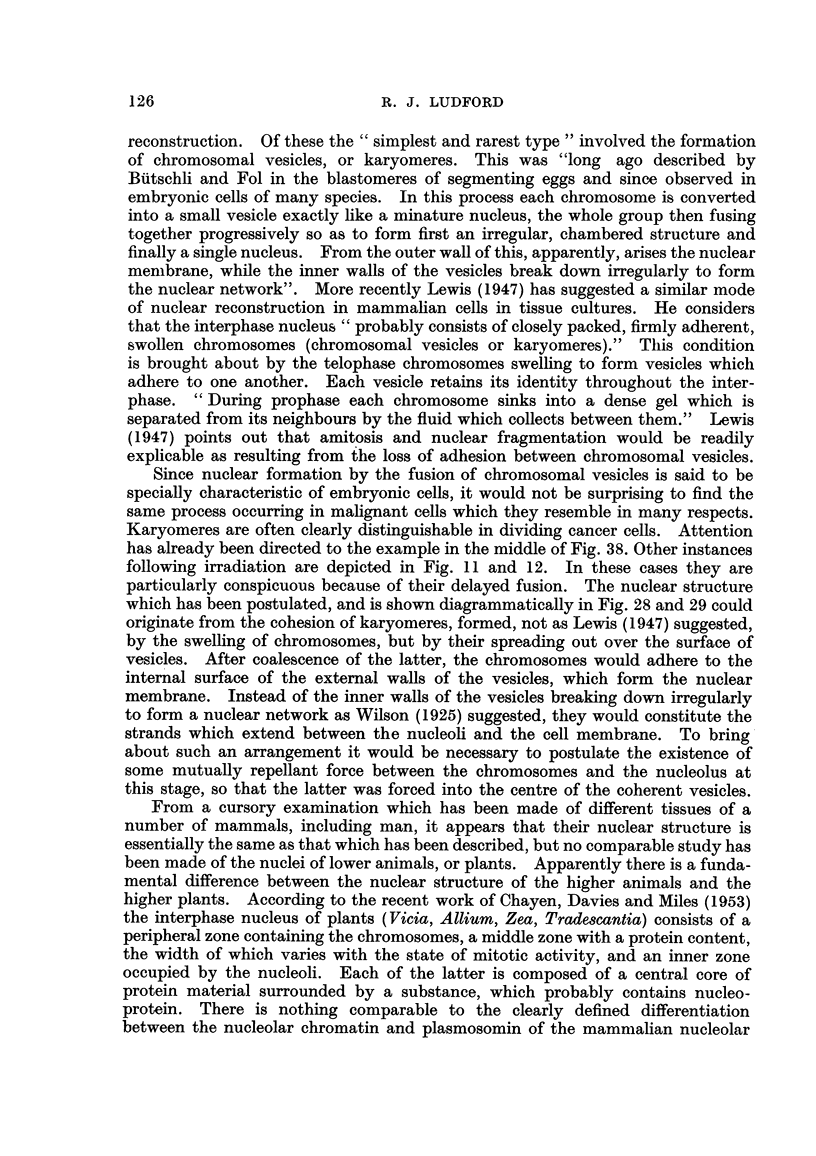

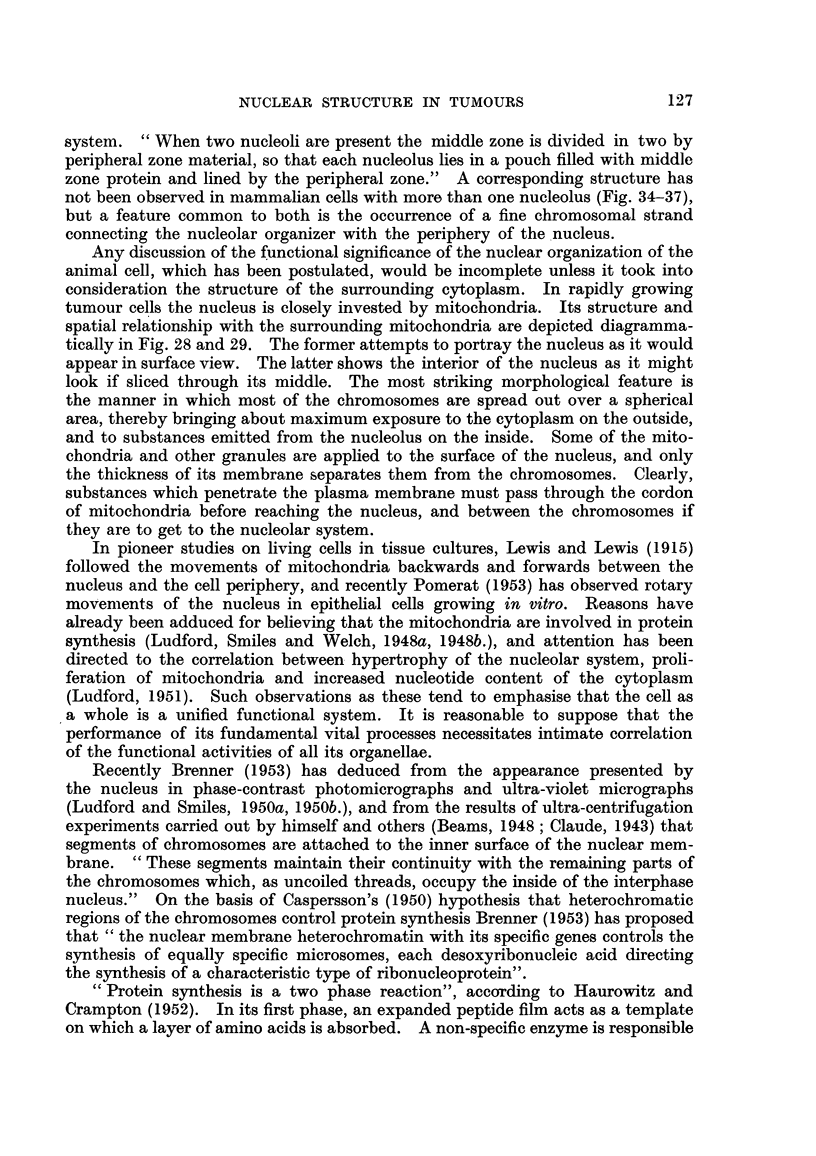

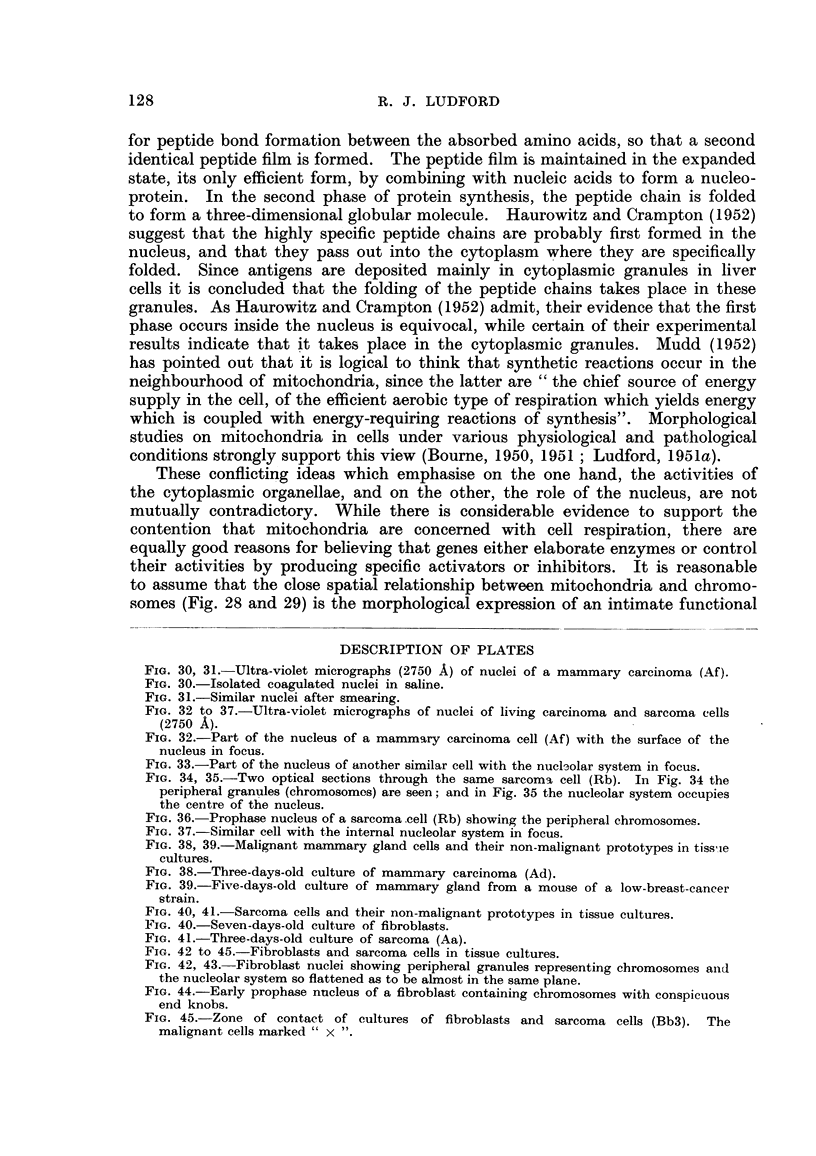

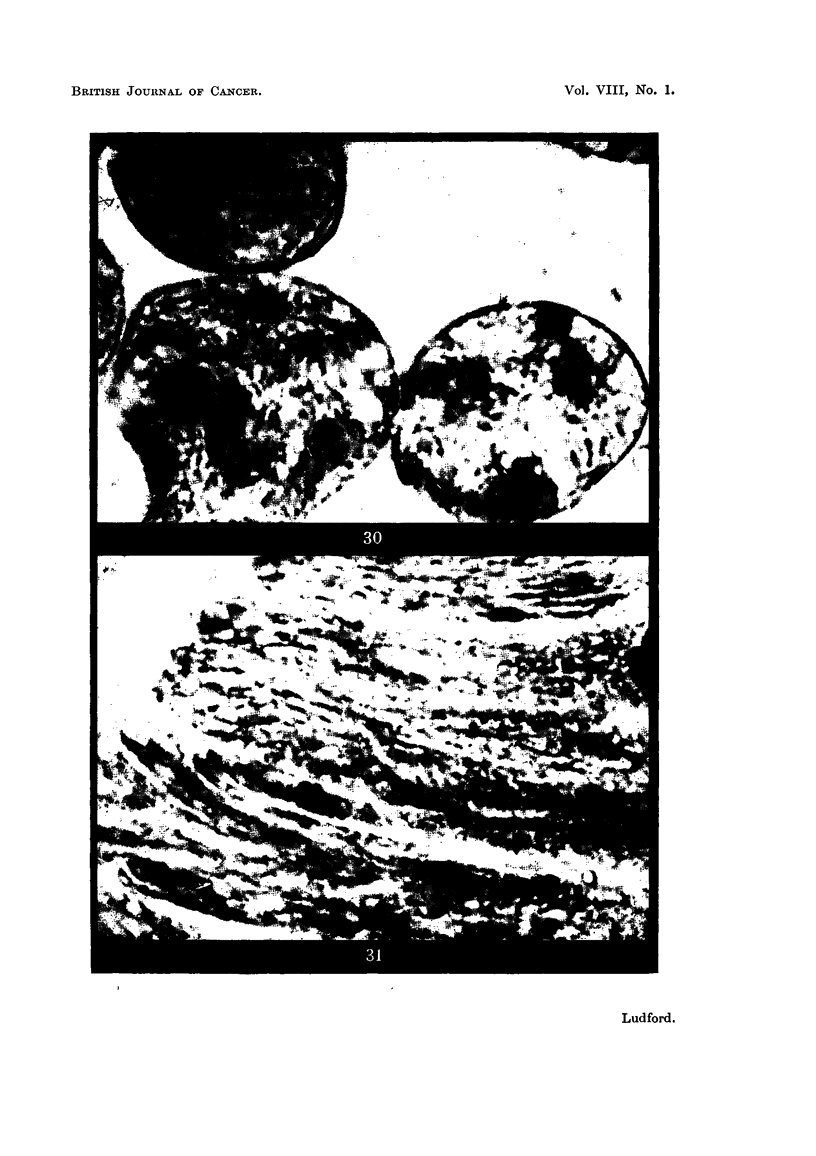

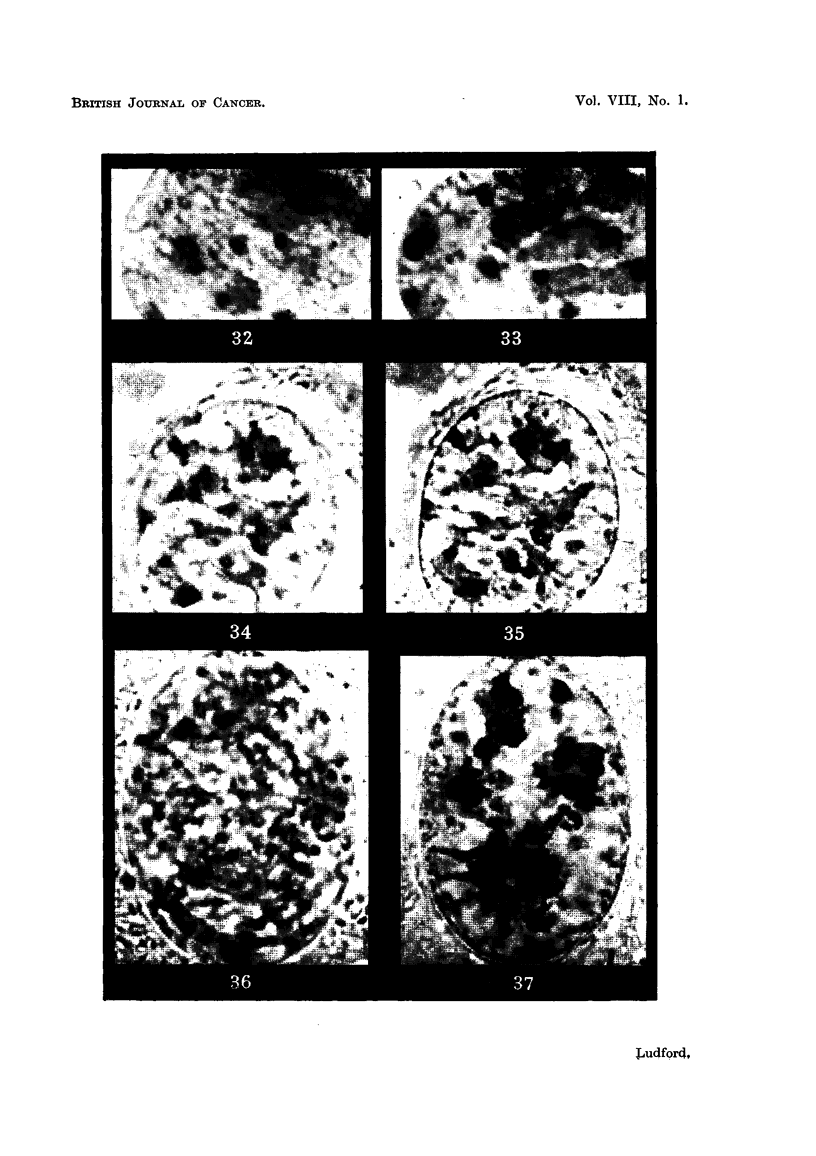

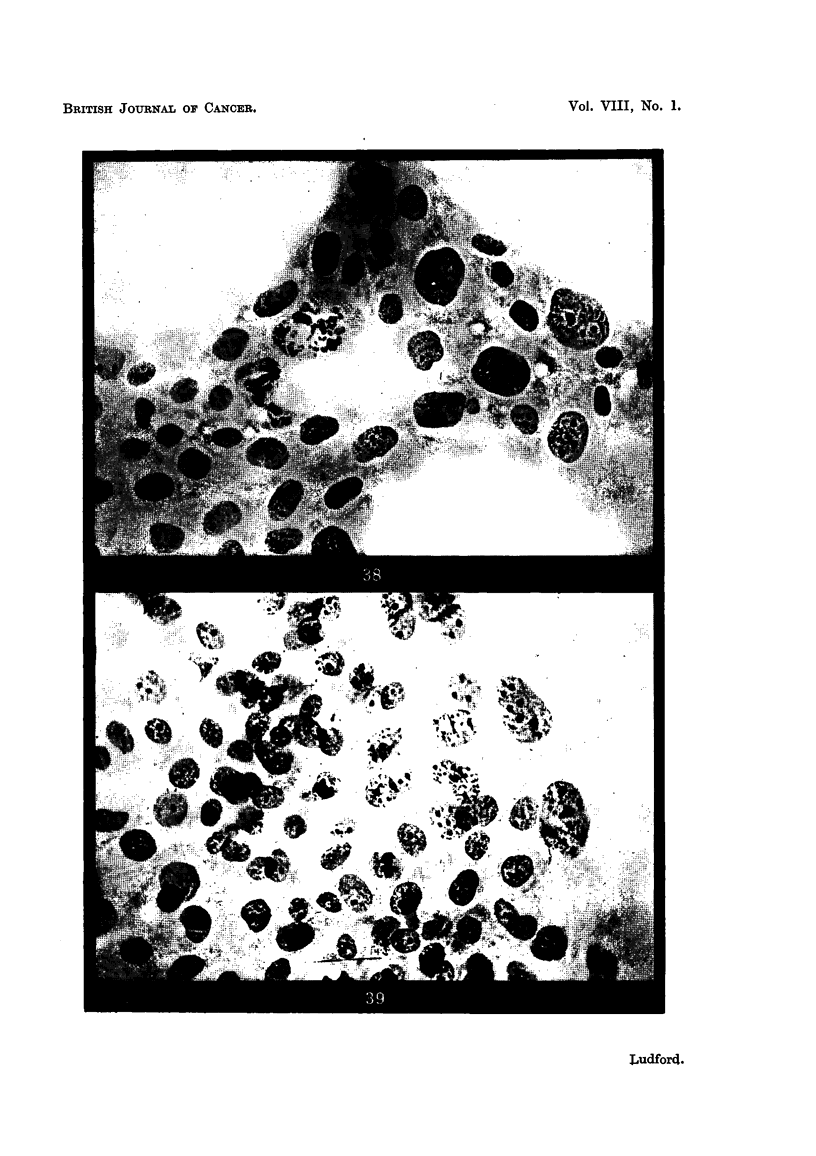

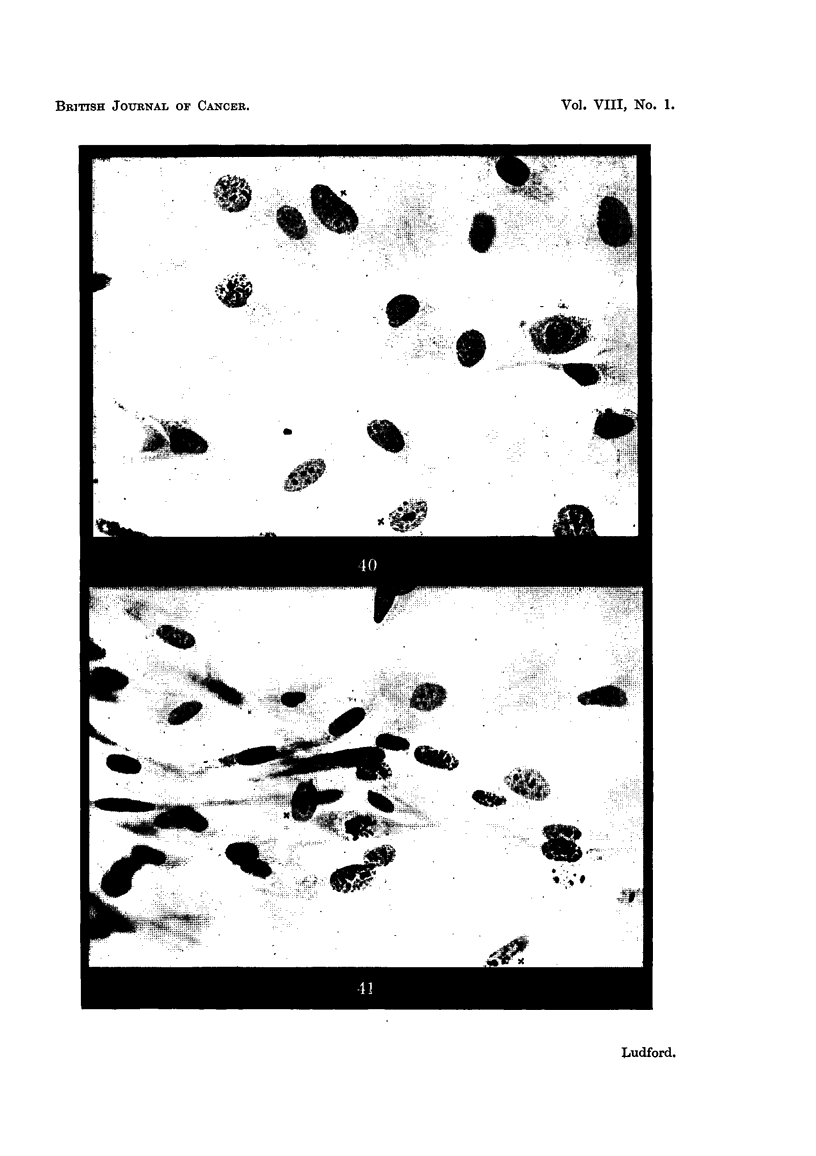

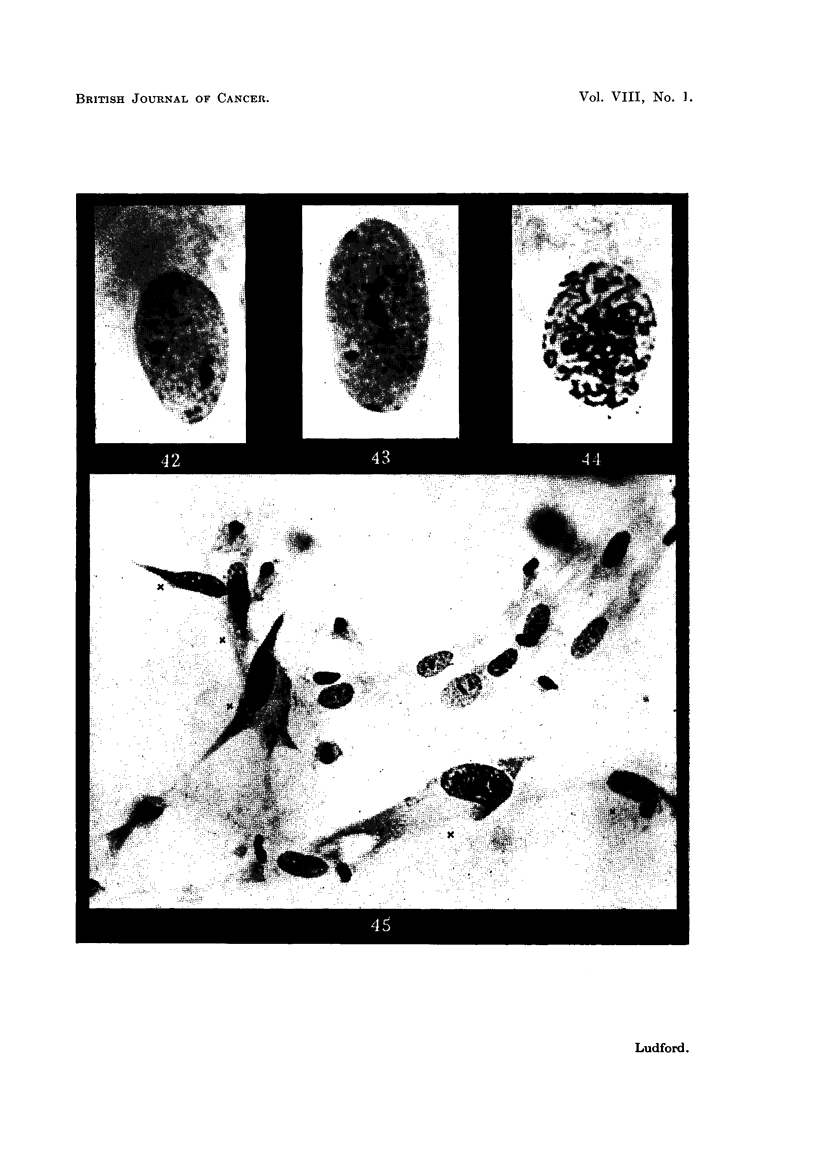

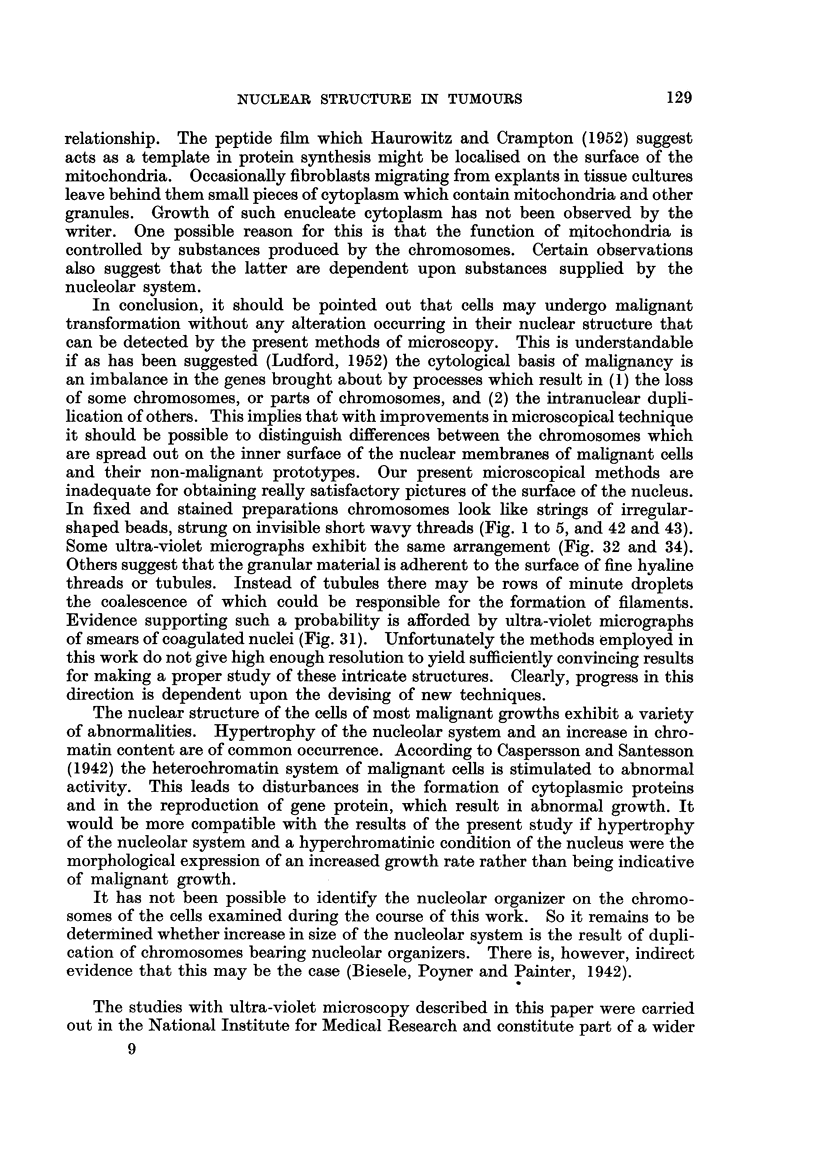

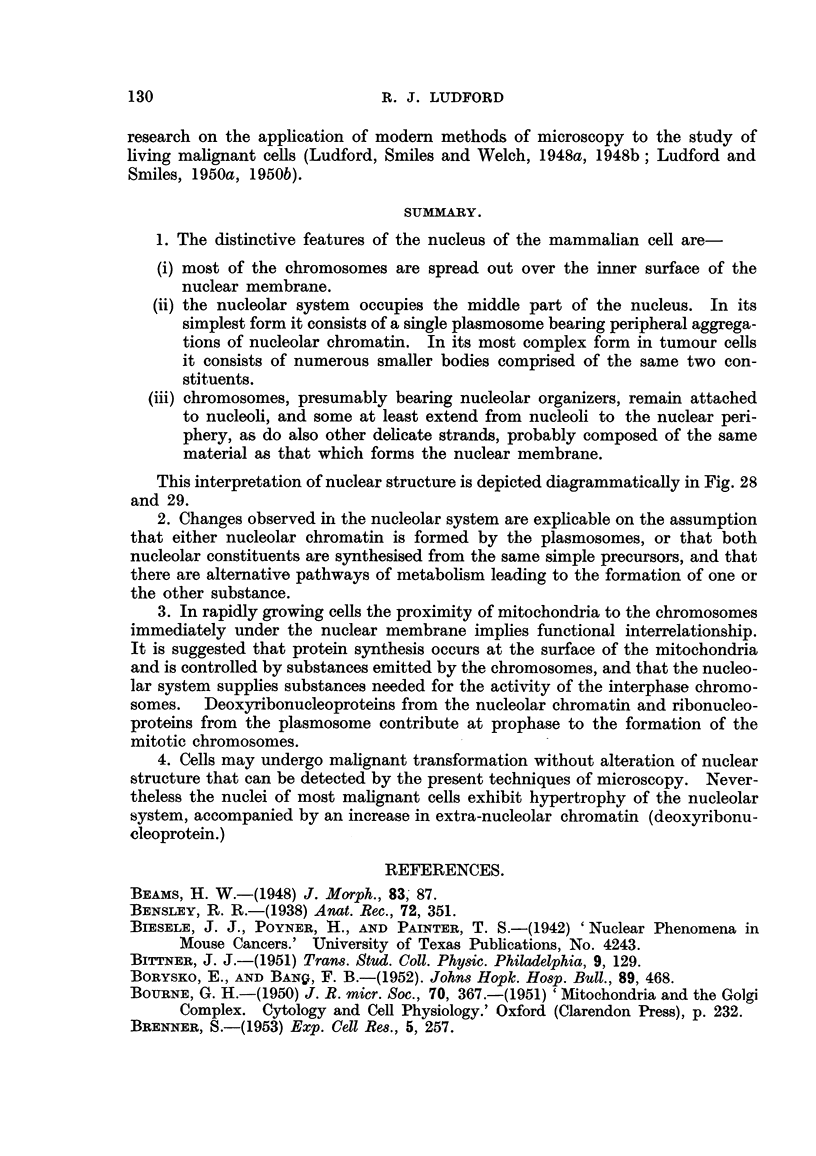

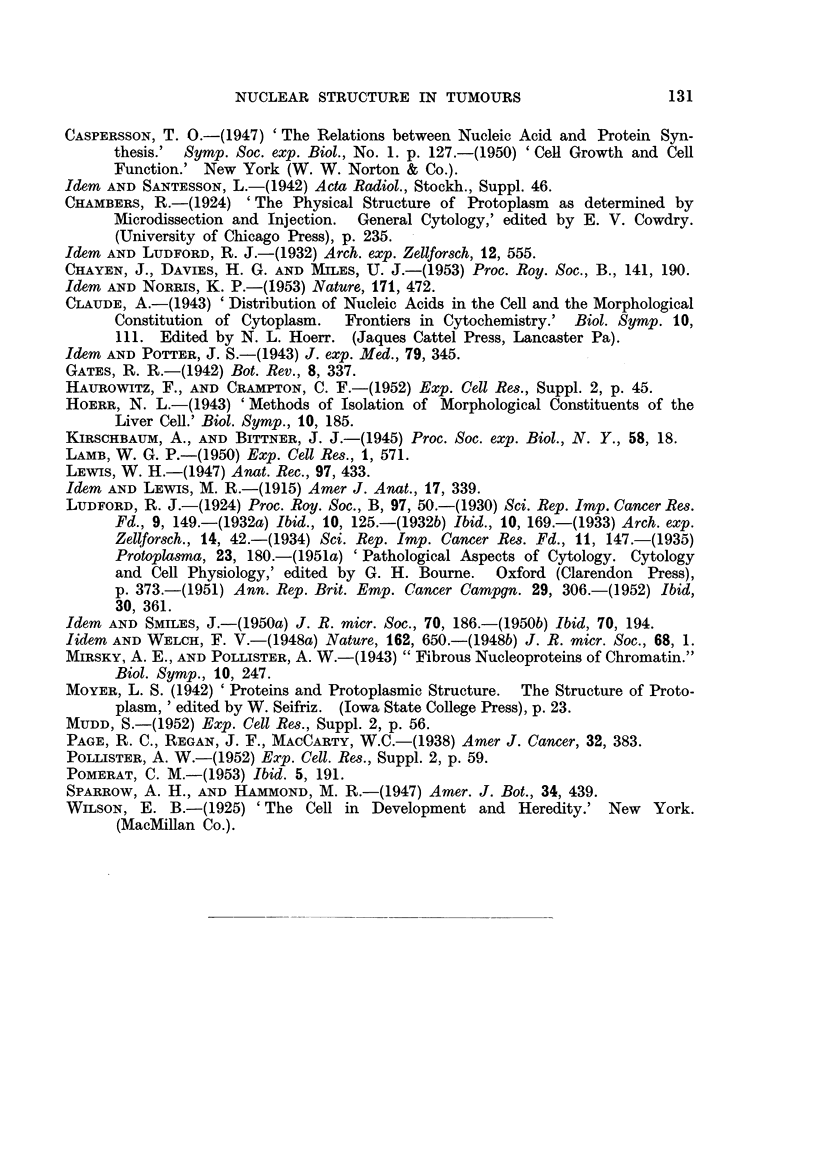


## References

[OCR_01247] BORYSKO E., BANG F. B. (1951). Structure of the nucleolus as revealed by the electron microscope; a preliminary report.. Bull Johns Hopkins Hosp.

[OCR_01252] BRENNER S. (1953). The chromatic nuclear membrane.. Exp Cell Res.

[OCR_01270] CHAYEN J., DAVIES H. G., MILES U. J. (1953). Observations on some plant interphase nuclei.. Proc R Soc Lond B Biol Sci.

